# Acute-on-chronic liver failure: consensus recommendations of the Asian Pacific association for the study of the liver (APASL): an update

**DOI:** 10.1007/s12072-019-09946-3

**Published:** 2019-06-06

**Authors:** Shiv Kumar Sarin, Ashok Choudhury, Manoj K. Sharma, Rakhi Maiwall, Mamun Al Mahtab, Salimur Rahman, Sanjiv Saigal, Neeraj Saraf, A. S. Soin, Harshad Devarbhavi, Dong Joon Kim, R. K. Dhiman, Ajay Duseja, Sunil Taneja, C. E. Eapen, Ashish Goel, Q. Ning, Tao Chen, Ke Ma, Z. Duan, Chen Yu, Sombat Treeprasertsuk, S. S. Hamid, Amna S. Butt, Wasim Jafri, Akash Shukla, Vivek Saraswat, Soek Siam Tan, Ajit Sood, Vandana Midha, Omesh Goyal, Hasmik Ghazinyan, Anil Arora, Jinhua Hu, Manoj Sahu, P. N. Rao, Guan H. Lee, Seng G. Lim, Laurentius A. Lesmana, Cosmas Rinaldi Lesmana, Samir Shah, V. G. Mohan Prasad, Diana A. Payawal, Zaigham Abbas, A. Kadir Dokmeci, Jose D. Sollano, Gian Carpio, Ananta Shresta, G. K. Lau, Md. Fazal Karim, Gamal Shiha, Rino Gani, Kemal Fariz Kalista, Man-Fung Yuen, Seema Alam, Rajeev Khanna, Vikrant Sood, Bikrant Bihari Lal, Viniyendra Pamecha, Ankur Jindal, V. Rajan, Vinod Arora, Osamu Yokosuka, Madunil A. Niriella, Hai Li, Xiaolong Qi, Atsushi Tanaka, Satoshi Mochida, Dominic Ray Chaudhuri, Ed Gane, Khin Maung Win, Wei Ting Chen, Mohd. Rela, Dharmesh Kapoor, Amit Rastogi, Pratibha Kale, Archana Rastogi, Chhagan Bihari Sharma, Meenu Bajpai, Virender Singh, Madhumita Premkumar, Sudhir Maharashi, A. Olithselvan, Cyriac Abby Philips, Anshu Srivastava, Surender K. Yachha, Zeeshan Ahmad Wani, B. R. Thapa, Anoop Saraya, Shalimar, Ashish Kumar, Manav Wadhawan, Subash Gupta, Kaushal Madan, Puja Sakhuja, Vivek Vij, Barjesh C. Sharma, Hitendra Garg, Vishal Garg, Chetan Kalal, Lovkesh Anand, Tanmay Vyas, Rajan P. Mathur, Guresh Kumar, Priyanka Jain, Samba Siva Rao Pasupuleti, Yogesh K. Chawla, Abhijit Chowdhury, Shahinul Alam, Do Seon Song, Jin Mo Yang, Eileen L. Yoon, APASL ACLF Research Consortium (AARC) for APASL ACLF working Party.

**Affiliations:** 1 Department of Hepatology, Institute of Liver and Biliary Sciences, New Delhi, 110070 India; 2 Department of Hepatology, Bangabandhu Sheikh Mujib Medical University, Dhaka, Bangladesh; 3 Department of Hepatology, Medanta The Medicity, Gurgaon, India; 4 Department of Hepatology, St John Medical College, Bangalore, India; 5 Department of Internal Medicine, Hallym University College of Medicine, Seoul, South Korea; 6 Department of Hepatology, PGIMER, Chandigarh, India; 7 Department of Hepatology, CMC, Vellore, India; 8 Institute and Department of Infectious Disease, Tongji Hospital, Tongji Medical College, Huazhong University of Science and Technology, Wuhan, China; 9 Translational Hepatology Institute Capital Medical University, Beijing You’an Hospital, Beijing, China; 10 Department of Medicine, Chulalongkorn University, Bangkok, Thailand; 11 Department of Medicine, Aga Khan University Hospital, Karachi, Pakistan; 12 Department of Gastroenterology, Lokmanya Tilak Municipal General Hospital and Lokmanya Tilak Municipal Medical College, Sion, Mumbai, India; 13 Department of Gastroenterology, SGPGIMS, Lucknow, India; 14 Department of Medicine, Hospital Selayang, Bata Caves, Selangor, Malaysia; 15 Department of Gastroenterology, DMC, Ludhiana, India; 16 Department of Hepatology, Nork Clinical Hospital of Infectious Disease, Yerevan, Armenia; 17 Department of Gastroenterology and Hepatology, Sir Ganga Ram Hospital and GRIPMER, New Delhi, Delhi India; 18 Department of Medicine, 302 Millitary Hospital, Beijing, China; 19 Department of Gastroenterology and Hepatology Sciences, IMS & SUM Hospital, Bhubaneswar, Odisha India; 20 Asian Institute of Gastroenterology, Hyderabad, India; 21 Division of Gastroenterology and Hepatology, Department of Medicine, National University Health System, Singapore, Singapore; 22 Digestive Disease and GI Oncology Centre, Medistra Hospital, Jakarta, Indonesia; 23 Department of Hepatology, Global Hospitals, Mumbai, India; 24 Department of Gastroenterology, VGM Hospital, Coimbatore, India; 25 Fatima University Medical Center Manila, Manila, Philippines; 26 Department of Medicine, Ziauddin University Hospital, Karachi, Pakistan; 27 Department of Medicine, Ankara University School of Medicine, Ankara, Turkey; 28 Department of Medicine, University of Santo Tomas, Manila, Philippines; 29 Department of Hepatology, Foundation Nepal Sitapaila Height, Kathmandu, Nepal; 30 Department of Medicine, Humanity and Health Medical Group, New Kowloon, Hong Kong China; 31 Department of Hepatology, Sir Salimullah Medical College, Dhaka, Bangladesh; 32 Egyptian Liver Research Institute And Hospital, Cairo, Egypt; 33 Division of Hepatobiliary, Department of Internal Medicine, Faculty of Medicine, Cipto Mangunkusumo Hospital, Universitas Indonesia, Jakarta, Indonesia; 34 Department of Medicine, Queen Mary Hospital Hong Kong, The University of Hong Kong, Hong Kong, China; 35 Department of Pediatric Hepatology, Institute of Liver and Biliary Sciences, New Delhi, Delhi India; 36 Department of Hepatobilliary Pancreatic Surgery and Liver Transplant, Institute of Liver and Biliary Sciences, New Delhi, Delhi India; 37 Professor Emeritus, Chiba University, Chiba, Japan; 38 Department of Medicine, University of Kelaniya, Ragama, Sri Lanka; 39 Department of Gastroenterology, Ren Ji Hospital, School of Medicine, Shanghai Jiao Tong University, Shanghai, China; 40 CHESS Frontier Center, The First Hospital of Lanzhou University, Lanzhou University, Lanzhou, China; 41 Department of Medicine, Tokyo University School of Medicine, Tokyo, Japan; 42 Department of Gastroenterology and Hepatology, Faculty of Medicine, Saitama Medical University, Saitama, Japan; 43 New Zealand Liver Transplant Unit, Auckland Hospital, Auckland, New Zealand; 44 Yangon GI and Liver Centre, Pabedan, Yangon, Myanmar; 45 Division of Hepatology, Department of Gastroenterology and Hepatology, Chang Gung Medical Foundation, Linkou Chang Gung Memorial Hospital, Taoyuan, Taiwan; 46 Department of Liver Transplant Surgery, Dr. Rela Institute and Medical Centre, Chennai, India; 47 Department of Microbiology, Institute of Liver and Biliary Sciences, New Delhi, Delhi India; 48 Department of Pathology, Institute of Liver and Biliary Sciences, New Delhi, Delhi India; 49 Department of Immunohematology and Transfusion Medicine, Institute of Liver and Biliary Sciences, New Delhi, Delhi India; 50 Department of Gatroenterology, SMS Med College, Jaipur, India; 51 Division of Liver Transplantation and Hepatology, Manipal Hospitals, Bangalore, India; 52 The Liver Unit, Cochin Gastroenterology Group, Ernakulam Medical Centre, Kochi, India; 53 Department of Pediatric Gastroenterology, SGPGIMS, Lucknow, India; 54 Noora Hospital in Srinagar, Shrinagar, India; 55 Department of Gastroenterology and Pediatric Gastroenterology, PGIMER, Chandigarh, India; 56 Department of Gastroenterology and Human Nutrition, AIIMS, New Delhi, India; 57 Department of Gastroenterology, Hepatology and Liver Transplant, B L K Hospital, New Delhi, India; 58 Centre for Liver and Biliary Science, Max Hospital, New Delhi, India; 59 Department of Gastroenterology, Hepatology and Liver Transplant, Max Hospital, New Delhi, India; 60 Department of Pathology, GB Pant Hospital, New Delhi, India; 61 Department of Liver Transplant and Hepatobilliary Surgery, Fortis Hospital, New Delhi, India; 62 Department of Gastroenterology, GB Pant Hospital, New Delhi, India; 63 Department of Gastroenterology, Hepatology and Liver Transplant, Apollo Hospital, New Delhi, India; 64 Department of Hepatology, Sir H N Reliance Hospital and Research Centre, Mumbai, India; 65 Department of Gastroenterology and Hepatology, Narayana Hospital, Gurugram, India; 66 Department of Hepatology, Parimal Multi-Speciality Hospital, Ahmedabad, India; 67 Department of Nephrology, Institute of Liver and Biliary Sciences, New Delhi, India; 68 Department of Statistics and Clinical Research, Institute of Liver and Biliary Sciences, New Delhi, India; 69 Department of Hepatology and Gastroenterology, Kalinga Institute of Med Sciences, KIIT University, Bhubaneswar, India; 70 Department of Hepatology, Institute of Post Graduate Medical Education and Research, Kolkata, India; 71 Department of Internal Medicine, College of Medicine, The Catholic University of Korea, Seoul, South Korea; 72 Department Of Internal Medicine, Inje University College of Medicine, Busan, South Korea

**Keywords:** Liver failure, Cirrhosis, Jaundice, AARC, Chronic liver disease, Alcoholic liver disease, ALF, Decompensation, Acute decompensation

## Abstract

The first consensus report of the working party of the Asian Pacific Association for the Study of the Liver (APASL) set up in 2004 on acute-on-chronic liver failure (ACLF) was published in 2009. With international groups volunteering to join, the “APASL ACLF Research Consortium (AARC)” was formed in 2012, which continued to collect prospective ACLF patient data. Based on the prospective data analysis of nearly 1400 patients, the AARC consensus was published in 2014. In the past nearly four-and-a-half years, the AARC database has been enriched to about 5200 cases by major hepatology centers across Asia. The data published during the interim period were carefully analyzed and areas of contention and new developments in the field of ACLF were prioritized in a systematic manner. The AARC database was also approached for answering some of the issues where published data were limited, such as liver failure grading, its impact on the ‘Golden Therapeutic Window’, extrahepatic organ dysfunction and failure, development of sepsis, distinctive features of acute decompensation from ACLF and pediatric ACLF and the issues were analyzed. These initiatives concluded in a two-day meeting in October 2018 at New Delhi with finalization of the new AARC consensus. Only those statements, which were based on evidence using the Grade System and were unanimously recommended, were accepted. Finalized statements were again circulated to all the experts and subsequently presented at the AARC investigators meeting at the AASLD in November 2018. The suggestions from the experts were used to revise and finalize the consensus. After detailed deliberations and data analysis, the original definition of ACLF was found to withstand the test of time and be able to identify a homogenous group of patients presenting with liver failure. New management options including the algorithms for the management of coagulation disorders, renal replacement therapy, sepsis, variceal bleed, antivirals and criteria for liver transplantation for ACLF patients were proposed. The final consensus statements along with the relevant background information and areas requiring future studies are presented here.

## Article Highlights

- Updated on the basis of AARC data of >3300 cases enrolled into AARC registry prospectively
- ACLF is distinct form Acute Decompensation of cirrhosis
- Newer sections on DILI-ACLF, AIH-ACLF, PVT/HVOTO–ACLF
- Reversibility of Chronic Liver Disease in ACLF
- Portal and systemic hemodynamics and their relevance in ACLF
- Acute Portal Hypertension and Variceal progression in ACLF
- AARC score as a guide for treatment strategies in ACLF
- ACLF in Children-first consensus on pediatric ACLF

## Introduction

Liver failure is a common medical ailment and its incidence is increasing with the use of alcohol and growing epidemic of obesity and diabetes. It can present as acute liver failure (ALF) (in the absence of any pre-existing liver disease), acute-on chronic liver failure (ACLF) (an acute deterioration of known or unknown chronic liver disease), or an acute decompensation of an end-stage liver disease [1, 2]. Each of these is a well-defined disease entity with a homogenous population of patients with expected outcomes. Due to an overlap and lack of clarity of definitions and outcomes, entities like late-onset liver failure, sub-acute hepatic failure, have become less relevant and there is lack of further publications suggesting removal of such terminologies to avoid confusion [1, 2].

The growing interest in ACLF after the first consensus definition of ACLF from APASL [2] is evident by the fact that more than > 450 publications as full papers have been published from the West (2) and the East and the trend is increasing. The group of investigators working on liver failure in the Asia–Pacific region working for the past decade carefully analyzed the patient characteristics, natural history and outcome of such patients. The group met on yearly basis and collated data on website (www.aclf.in). With the setting up of the APASL ACLF Research Consortium (AARC) in 2012, the collaborative research work, publications and protocol driven unified treatment had gained momentum. The retrospective and prospective data of patients from different centers were analyzed, and the completed patient records were utilized for defining predictors of mortality and grades of liver failure and incidence of other organ failures [3].

The APASL ACLF consensus of 2014 was based on about 1363 patients from 14 countries. During the past nearly four and a half years (2014–2018), 5228 patients of 43 Centers from 15 countries have so far been registered in the AARC database. These patients have been prospectively enrolled and followed and form the basis of the new structured consensus.

Experts from across the world, especially from the Asia–Pacific region, were requested to identify pertinent and contentious issues in ACLF. After a round of deliberations, 8 major issues were identified for update. Further, data from the AARC database were taken and analyzed and circulated to all the participants.

The process for the development of the recommendations and guidelines included: review of all available published literature on ACLF by individual and group of experts; preparation of a review manuscript and consensus statements based on GRADE SYSTEM (Table 1) of evidence-based approach [4], circulation of consensus statements to all experts, a survey of the current approaches for the diagnosis and management of ACLF; discussion on contentious issues; and deliberations to prepare the consensus statement by the experts of the working party. A 2-day meeting was held on October 1–2, 2018, at New Delhi, India, to discuss and finalize the consensus statements, recommendations and guidelines. The finalized statements were circulated to all the experts and subsequently finalized. These consensus statements and recommendations for the diagnosis and management of ACLF are included in this review. A brief background is included providing the available data and published information on each of the issues. Statements from the previous consensus have been reproduced at places to give a background and continuity.

**Table 1:** Evidence grade used for the APASL Guidelines Adopted from Atkins et al. [4]

Grading of evidence	Notes	Symbol
High quality	Further research is very unlikely to change our confidence in the estimate effect	A
Moderate quality	Further research is likely to have an important impact on our confidence in the estimate of effect and may change the estimate effect	B
Low or very low quality	Further research is very likely to have an important impact on our confidence in the estimate of effect and may change the estimate effect. Any estimate of effect is uncertain	C
Grading of recommendations	Notes	Symbol
Strong recommendation warranted	Factors influencing the strength of the recommendation included the quality of the evidence, presumed patient-important outcomes, and cost	1
Weaker recommendation	Variability in preferences and values, or more uncertainty: more likely a weak recommendation is warranted. Recommendation is made with less certainty: higher cost or resource consumption	2

### The concept of ACLF and hepatic reserve

Acute liver failure is a well-defined medical emergency which is defined as a severe liver injury, leading to coagulation abnormality usually with an INR ≥ 1.5, and any degree of mental alteration (encephalopathy) in a patient without pre-existing liver disease and with an illness of up to 4 weeks duration [5].A proportion of patients who present with features mimicking ALF, however, have an underlying chronic liver disease or cirrhosis of the liver. These patients grouped together as acute-on-chronic liver failure (ACLF) also have a poor outcome. These patients are distinctly different from a group of cirrhotic patients who are already decompensated [6] and have a sudden worsening of their condition, i.e., acute decompensation (AD) due to an acute event that may present with hepatic or non-hepatic failure [6].

ACLF is a clinical syndrome manifesting as acute and severe hepatic derangements resulting from varied insults. This term was first used in 1995 to describe a condition in which two insults to liver operate simultaneously, one of them being ongoing and chronic and the other acute [7]. Over the years, several definitions have been proposed, creating confusion in the field [8]. The time frame for the development of liver failure and ACLF has been several times changed from 12 to 4 weeks again to 12 weeks [9]. Moreover, the nature of insult and the stage of underlying disease have been variably used.

**Fig. 1:**
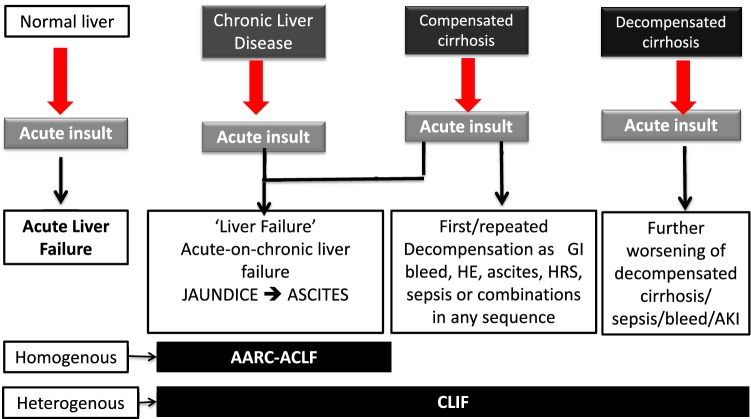
Concept of ACLF and the cohorts included in different definitions. The figure describes the response of the liver to an acute hepatic injury, depending on the underlying hepatic injury, prior decompensation, time frame from insult to presentation with decompensation and reversibility with mitigation of the acute insult. The spectrum extends from acute liver failure, acute-on-chronic liver failure, acute decompensation, end-stage liver disease. ACLF is distinct like ALF when the APASL definition is considered. APASL definition is simple and homogenous and is distinct

In fact, any patient who has an underlying chronic liver disease with superimposed acute insult is labeled as having ACLF, irrespective of evidence of liver failure *per se* or evidence of pre-existing cirrhotic decompensation. Several investigators were concerned that this would lead to substantial overlap with decompensated liver disease. The main emphasis of the fourth consensus meeting of the APASL Working Party was to identify from this large group of patients, a subset of patients who have a relatively homogenous presentation and potentially similar outcome and restrict the use of the term ‘‘acute-on-chronic liver failure’’ to this subset. The 2009 APASL definition had provided a basis to select patients presenting with a distinct syndrome. To cover the entire spectrum of these patients, from mild to most severe, patients with chronic liver disease with or without cirrhosis of the liver were included and carefully analyzed. It is understandable, though not well defined, that the nature and degree of acute insult and the status of the underlying chronic liver disease would determine the outcome in a patient (Fig. 1).

To give clarity to the primary event, a hepatic insult, jaundice and coagulopathy, which defined liver failure, was considered essential. In acute liver failure, though hepatic encephalopathy (HE) is part of the definition, it follows liver failure. Should one wait for defining the outcome of ‘acute liver failure’ till the time extrahepatic organ failures set in or not, remains contentious. For definition, the event must be universally present in all patients. From the point of view of intensivists, it is well known that with increasing number of organ dysfunction or failure, the mortality would cumulatively increase. Undoubtedly, these events are predictive of the outcome, the basis of SOFA score [10]. It is, therefore, not surprising; the same has been reported in the Western studies [11]. However, should organ failure be included in defining the clinical syndrome of liver failure needs a thorough analysis. As a corollary, despite decades of extensive experience, renal or circulatory dysfunction has not been included in the definition of ALF. The issue whether sepsis *per se* could lead to liver failure or is a result of liver failure had been debated for many years and was again revisited. However, sepsis is an integral part of development of multi-organ failure in any patient, be it of renal, pancreatic or cardiac origin.

In essence, ACLF is a distinct entity where acute hepatic decompensation occurs in an established chronic liver disease or cirrhosis patient on exposure to acute insult in a defined time frame resulting in a high short-term mortality. Based on the data, attempts were made if the current definition of ACLF could be improved further (Table 2). Five aspects were worked upon:

**Table 2:** Comparison of the existing ACLF definitions commonly accepted

	APASL	EASL/CLIF	NASCELD
Definition	Acute hepatic insult manifesting as jaundice and coagulopathy Complicated within 4 weeks by ascites and/or encephalopathy in a patient with previously diagnosed or undiagnosed chronic liver disease associated with high mortality.	An acute deterioration of pre-existing chronic liver disease usually related to a precipitating event and associated with increased mortality at 3 months due to multisystem organ failure	A syndrome characterized by acute deterioration in a patient of cirrhosis due to infection presenting with two or more extrahepatic organ failure.
Study cohort	First consensus was the expert opinion, subsequently prospectively evaluated in 1402 patient, subsequently in 3300 patients.	Prospectively studied in 1343 patients	Prospectively studied in 507 patients
Inclusion	Compensated Cirrhosis (diagnosed or non-diagnosed) CLD but not cirrhosis Acute insult directed to liver Presentation with liver failure to start with Index presentation	Cirrhosis only Compensated or decompensated Renal failure is mandatory (not liver failure for defining ACLF) Presentation not necessarily be liver failure Can be repeated episodes ACLF	Cirrhosis only Compensated or decompensated Two extrahepatic organ failure Presentation not necessarily be liver failure Can be repeated episodes of ACLF
Diagnosis	Early, reversibility is likely and thus may affect outcome	Too late, reversibility is unlikely and thus may not affect outcome	Too late, reversibility is unlikely and thus may not affect outcome
Exclusion Criteria	Prior decompensation HCC	HCC	Patients who had infections but did not require hospital admission. Cirrhosis without infection. Immune-compromised patients with human immunodeficiency virus (HIV) infection, prior organ transplant, and disseminated malignancies
Homogeneity	Yes. Index presentation, previously unknown or compensated, acute hepatic insult leading to liver failure as the driver.	No. Any presentation, with prior decompensation or recent worsening of ongoing decompensation, acute insult is not directed to liver, in particular (40% are of unknown acute insult), not liver but extrahepatic organ failure, i.e., renal failure is must, systemic inflammation but not the liver as driver.	No. Any presentation, with prior decompensation or recent worsening of ongoing decompensation, acute insult is not directed to liver in particular Any extrahepatic organic failure
Time frame	4 weeks	4–12 weeks (variable)	Not defined
Acute insult	Hepatic	Hepatic or Systemic (extrahepatic)	Infection, i.e., systemic (extrahepatic)
Sepsis	Consequence/complication	Cause/precipitant	Cause/precipitant
Organ failure	Liver is primary to start with Others subsequently	Systemic inflammation leading to kidney failure as the primary with or without other organ failure	Systemic inflammation leading to extrahepatic organic failure
Disease severity score	AARC Score-prospective as well as validated	CLIF-C SOFA, Prospective but only expert opinion	MELD CLIF-C SOFA
Golden window	Well defined for therapy, i.e., by 7 days SIRS or sepsis as well as for decision regarding Liver Transplant	No such	No such
Pediatric cohort	Yes	None	None
Therapy	Regenerative and bridging therapy with good result	No such	No such
Reversibility of ACLF syndrome	Yes	Not described	Not described

1. *The time frame for the acute insult* in the initial (2009) and subsequent definition of ACLF, the time for development of ascites and/HE after appearance of jaundice and coagulopathy was kept as 4 weeks (28 days) [1, 2]. A mortality rate of more than 33% at 4 weeks was considered to be significant allowing recovery to less than two-third of the patients in the 2014 consensus. The additional new data after the previous consensus were carefully analyzed and it showed a 4-week mortality of around 39.9% [2]. Therefore, the definition of 4 week for acute insult in ACLF was considered as appropriate and was maintained.
2. *Reversibility of the ACLF syndrome* this is a feature of the ACLF defined by the AARC criteria, as nearly all the patients included are after the index presentation. With mitigation of acute insult and over time, the hepatic reserve improves, fibrosis regresses and the portal pressure decreases. It was decided to define reversibility as reversal of key components that were used for defining the syndrome of liver failure, i.e., decrease of bilirubin below 5 mg/dL and reversal of coagulopathy to INR below 1.5 and no encephalopathy with or without resolution of ascites. It was interesting to find in the large AARC database, of the 1844 patients with complete data until day 90. About 70% of the survivors beyond day 90, showed reversibility and they maintained this status for a period of at least 1 year. Further, unlike patients with decompensated cirrhosis and similar to patients with ALF, the reversal of coagulopathy preceded the reversal of jaundice, i.e., median time to reversal of coagulopathy was 7 (4–30) days versus 19 (7–60) days for jaundice, respectively. The median time to reversal of syndrome, i.e., jaundice and coagulopathy, was 30 days. Baseline albumin, AARC score and transient elastography predicted long-term reversibility in the recently analyzed AARC data.
3. *Recording ‘Index ‘or first presentation in the definition of ACLF* this issue was deliberated so as to define and include a homogenous cohort of patients. The consideration of prior decompensation with recent worsening (difficult to differentiate from acute decompensation, AD) or recovery from ACLF and followed by subsequent presentation as ACLF (i.e., ‘ACLF again’) will lead to confusion. It is important to distinguish the syndrome of ACLF from other forms of liver failure, such as acute decompensation and end-stage liver disease (ESLD). There was a consensus to initiate prospective studies comparing patient manifesting with index presentation, prior decompensation or recent worsening of decompensated cirrhosis patients.
4. *Inclusion of mortality in definition* the mortality was included in 2014 AARC consensus definition for identifying a set of patients who have high 28-day mortality so as to prioritize them for admission, treatment and liver transplantation. At present, the ACLF definition both by the APASL and CLIF-EASL includes mortality. The group of experts raised the concern that mortality is generally not part of definition in disease conditions. Other experts, however, disagreed to this. After due deliberations, it was decided to keep the statement on mortality, in the AARC-ACLF definition.
5. *Inclusion of organ failure in definition* the Western definitions of ACLF include organ failure in the definition. This issue was debated extensively. The data from the AARC database were also analyzed. It was reiterated that organ failure other than liver should not be part of the definition. Diagnosis of liver failure along with kidney, circulatory and respiratory failure is generally a late event and is often a result of the primary organ, i.e., liver failure (jaundice, deranged coagulation and/or HE). The experts felt that organ dysfunction rather than organ failure should be the time for raising suspicion and making diagnosis of ALCF rather than when organ failure(s) has already developed. This approach would help in prioritizing organ-specific interventions.

The AARC definition of ACLF is a simple bed-side tool (requires history taking, physical examination and simple laboratory parameters) and can be used by primary care givers. It enables a clinician to stratify a patient presenting with liver failure for early referral, early intervention and, hence, allows a better chance of reversibility as well as improved survival. The earlier criteria for defining the nature of acute insult were reiterated, i.e., the event must be new and acute and its impact on the patient’s condition should be discernible as liver failure within a given time frame of 4 weeks [1, 2].

**Recommendations**

1.0 *The concept of ACLF and hepatic reserve.*
1.1 The 28- and 90-day mortality is high in ACLF patients (A1).
1.2. Among the survivors at day 90, the reversal of ACLF syndrome was noted in nearly 70% cases (C2).
1.3. Almost two-third of the patients, who had reversal of the ACLF syndrome by day 90, show a persistent regression of the disease at 1 year (C2).
1.4. Reversal of coagulopathy precedes the reversal of jaundice (C2).
1.5. The baseline AARC liver failure grade can identify patients who are likely to reverse (C2).
1.6. A higher platelet count, lower leukocyte count and the absence of HE are additional independent predictors of reversibility (C2).
1.7. Transient elastography needs to be evaluated for identifying the reversibility of ACLF syndrome at baseline as well as at follow-up (C2).
1.8 *Will inclusion of terminology of ‘First’ presentation in definition improve clarity and homogeneity.*
1.8.1. Inclusion or exclusion of prior decompensation and ‘first’ presentation for the definition of ACLF needs prospective studies [B2].
1.9. *Including organ failure in definition- for utility or futility?*
1.9.1. The terms “organ dysfunction” and “organ failure” need to be described more clearly based on the AARC database, used in APASL consensus [B2].
1.9.2. Extrahepatic organ failure should not be included in definition of ACLF, as this would lead to missing out the potential therapeutic window for reversal of the ACLF syndrome (A1).
1.9.3. Liver failure for definition of ACLF should include jaundice (serum bilirubin ≥ 5 mg/dL) and coagulation dysfunction (INR > 1.5) (A1).
1.10. *Whether mortality should be part of definition?*
1.10.1. Mortality should not be part of the definition of ACLF. One need not die to fulfill the criteria of ACLF definition. Mortality is not generally part of any definition in disease conditions (C2). However, since the earlier (2014) definition had included mortality, the same definition was agreed.

### Definition of ACLF

There is no consistent definition of ACLF in the literature. Each study done previously on ACLF has used its own definition, and there is no unanimity in these definitions in terms of criteria for liver failure, the acute event precipitating ACLF, and the diagnosis of underlying chronic liver disease. Since most of these studies were on patients who required liver support devices or liver transplantation, these studies were biased toward including sicker patients in the definition and patients having a mild disease were left out.

A detailed analysis of the definition of liver failure and the need for the defined outcome of high 28-day mortality was taken into account. An estimated 33% mortality at 28 days was considered important. Having analyzed and defined the acute and chronic insults, the time frame and the criteria of liver failure, development and course of organ failure and sepsis, the APASL definition of ACLF of 2009 was reassessed. It was reported that this definition has been used in nearly 200 publications from the East and the West and has been found to be simple to use and with a high degree of predictive ability to define the outcome of a relatively homogenous group of liver failure patients with underlying chronic liver disease.

The consensus definition is:

- “ACLF is an acute hepatic insult manifesting as jaundice (serum bilirubin ≥ 5 mg/dL (85 micromol/L) and coagulopathy (INR ≥ 1.5 or prothrombin activity < 40%) complicated within 4 weeks by clinical ascites and/or encephalopathy in a patient with previously diagnosed or undiagnosed chronic liver disease/cirrhosis, and is associated with a high 28-day mortality.” (I, A).

#### Defining the acute insult

The spectrum of acute insult in the Asian region was revisited, while hepatitis B reactivation remains the predominant cause of acute hepatic insult in the East, from the global perspective. The trends showed an increase in the proportion of alcoholic hepatitis over the years. This was a bit unexpected for the Asian countries where alcoholic hepatitis is emerging as a major acute insult and shows the growing westernization of Asia. A review of the recent CANONIC study data showed that in the West the term precipitating event is generally used and probably details of events such as Hepatitis B or superadded hepatitis A and E are rarely encountered or recorded [11]. Surprisingly, even the active alcohol abuse and alcoholic hepatitis were also not the predominant causes. A plausible explanation could be that since the CANONIC study only recorded the acute decompensation of cirrhosis and not the hepatic insults, the major events recorded were only non-hepatic, such as bacterial infections or sepsis. Acute decompensation of cirrhosis is a different entity than ACLF. As the core premise of ACLF is presentation as liver failure, the acute insults should be hepatic insults. Both hepatotropic and non-hepatotropic insults should manifest in the patient first with liver failure.

**Fig. 2:**
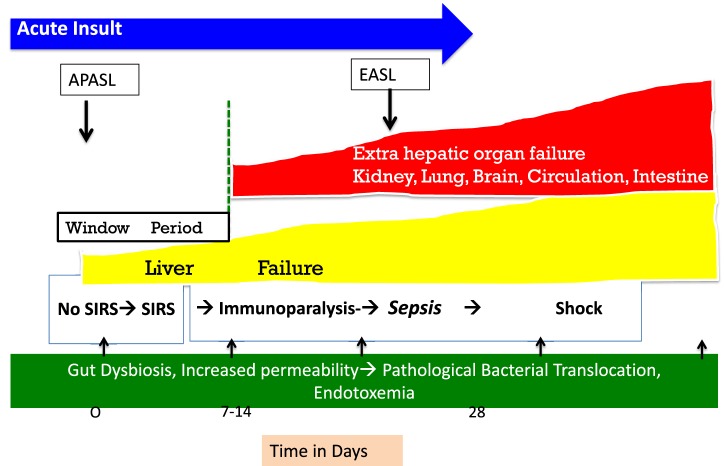
Sequence of events in Diagnostic Criteria of ACLF: East vs. West. The figure clearly describes the sequence of organ failure and its mechanism. An acute hepatic insult leading to hepatic decompensation is the driver and subsequent extrahepatic organ failure is due to failure of recovery/regeneration and development of sepsis after a Golden Window. With consideration of sepsis as the intiating factor and development of extrahepatic organ involvement as a part of definition leads to late identification of the ACLF patients where the therapeutic windos is lost. The difference between ACLF, AD and ESLD (as in Fig. 1) is blurred and entity is heterogenous. So pure hepatic insult leading to hepatic failure at the beginning and subsequent extrahepatic organ failure as complication, not defining complex is the crux in managing this group of liver disease patient

Acute hepatic insults of infectious etiology included reactivation of hepatitis B virus (HBV) as the leading cause of ACLF in the Asian region [12–20]. Reactivation of HBV could be either spontaneous or due to intensive chemotherapy or immunosuppressive therapy [12, 13], immune restoration after highly active antiretroviral therapy for HIV [14, 15], treatment related [16], or reactivation of the occult HBV infection by rituximab (anti-CD20)-based chemotherapy [17–19]. Similarly, reactivation of hepatitis C virus infection has also been reported, especially after immune suppressive therapy [20, 21]. The other very important infectious etiology of the acute event is super-infection with hepatitis E virus, predominantly in patients in the Indian subcontinent [22–25]. Various bacterial, parasitic, and fungal infections may affect the liver. Spirochetal, protozoal, helminthic, or fungal organisms may directly infect the liver, whereas bacterial or parasitic infection may spread to the liver from other sites [26]. These infections may lead to liver failure in patients with underlying chronic liver disease. Among the non-infectious etiologies, alcoholic hepatitis is the major cause of acute deterioration in stable known or unknown chronic liver diseases, more often in the western countries [27, 28]. It was not clear what should be the interval from the last alcoholic drink to be included as acute insult. Since, after the direct hepatic injury, the immunological injury starts to decline [29], a period of 28-day was considered adequate for inclusion as the last drink. The issue, which remained to be addressed, was of binge drinking in patients with ACLF due to recent alcohol intake. It was appreciated that a prospective data collection including the drinking behavior especially in the past 6 months would help decide the influence of drinking behavior on the clinical outcome and help in defining the time frame of what should be considered as an acute insult (Fig. 2).

(A) *Drug-induced liver injury (DILI*) presenting as ACLF is an important entity less often addressed in the Global literature. Hepatotoxic drugs and complementary and alternative medicines (CAM) are important causes for acute and acute-on-chronic liver failure in the Asia–Pacific region [30]. While most drugs are safely tolerated in the setting of CLD, recent work suggests that individuals with CLD may be at increased risk to develop hepatotoxicity at least to certain drugs [31]. Hepatitis following the use of anti-tubercular drugs was considered to be an important cause of acute insult leading to ACLF. In a proportion of patients, despite a history of use of CAM, the precise nature and injurious influence of the agent cannot be determined. Results from the drug-induced liver injury network have demonstrated that mortality in 89 patients with pre-existing liver disease was 16% which was significantly higher than the 5% mortality in 810 patients with underlying liver disease [32]. Drugs such as anti-tuberculosis drugs, methotrexate and antiretroviral drugs in HIV/AIDS-infected individuals have been implicated as triggering liver injury include particularly in the setting of underlying chronic liver disease due to hepatitis B or C [33–37]. Paradoxically CLD or cirrhosis is a risk factor for tuberculosis [38, 39] and first-line anti-tuberculosis drugs have been consistently shown to increase the risk of hepatotoxicity, particularly in hepatitis B and C [40, 41]. Although drugs have been listed as a precipitant factor in ACLF, data are scarce except from the APASL/AARC database. Data from the West is lacking on drugs as an acute insult leading to ACLF. In Asia 1.8% [42] to 5.7% [43, 44] precipitating events for ACLF are related to drugs. In Chinese cohort, the drugs were mostly from herbal or traditional medications to anti tuberculosis drugs in Indian cohorts [44]. From the AARC database, 329 (10%) of the 3132 patients of ACLF had an inciting event due to drugs. There is, however, need for further data and work on the modes of hepatic injury caused by different herbal and medicinal preparations on patients with cirrhosis.

(B) *Autoimmune hepatitis flare* leading to ACLF has not been adequately addressed in both Asia Pacific as well as Western cohorts. The pattern of clinical presentation spans from benign chronic hepatitis and indolent disease to acute liver failure. The abrupt presentation can indicate spontaneous exacerbation of a pre-existent chronic disease (presenting as ACLF), newly developed disease (presenting as ALF), a superimposed infectious or toxic injury, or new disease after viral infection, drug therapy, or liver transplantation. Approximately, 20% of patients with autoimmune hepatitis present with severe jaundice, HE and coagulopathy, with or without ascites, which resemble ALF or ACLF [45]. The disease usually has an unusual presentation with nearly half the patients being seronegative, requiring a lower threshold for transjugular liver biopsy. The histological features are distinct from those found in fulminant AIH. Stravitz et al. [46] identified lymphoid aggregates, perivenulitis and massive hepatitic necrosis as suggestive histological features of AIH–ALF [47]. The multicentric AIH-ACLF data from AARC database, which showed that the lymphoid aggregates and perivenulitis are less common in AIH-ACLF. However, advanced fibrosis (F3/F4), ductular reactions, and large areas of parenchymal collapse with lymphoplasmacytic inflammation are prominent findings along with classical autoimmune features in AIH-ACLF. It was observed that use of steroid in a select group of moderately severe AIH has a favorable outcome. Autoimmune hepatitis (AIH) was the first liver disease for which an effective therapeutic intervention was provided and treatment efficacy shown [47].

(C) *Acute variceal bleeding* has been included as one of the events to define acute decompensation in the natural history of cirrhosis [48]. Variceal bleeding has also been taken as an acute insult for ACLF in some western trials of ACLF. A scenario may exist that a patient who has already fulfilled the criteria of ACLF and has been diagnosed ACLF, develops a variceal bleed. In such a patient, variceal bleed would be considered as a complication in the natural history of ACLF. In recently analyzed 1028 compensated cirrhosis patients presenting with acute variceal bleed, the syndrome of ACLF was seen in 4% cases. Acute variceal bleed led to 10% mortality in compensated cirrhosis, which increased to 18% in 90-day follow-up with the development of ACLF (*p* < 0.001). A large set of data was mined and the issue was debated whether to consider variceal bleed as an acute event of ACLF. However, since the definition of ACLF includes liver failure, jaundice and coagulopathy, the variceal bleed should result in liver failure. The liver failure in such patients is likely to be due to hepatic ischemia [49] and subsequent bacterial infections [50]. It was discussed that for a patient with portal hypertension and cirrhosis of the liver who presents for the first time with variceal bleed without any previous or present signs or symptoms of chronic liver disease, it would not constitute an acute insult. This is especially relevant if such a patient does not develop any jaundice. The experts discussed the stratification of patients based on the stage of underlying liver disease and the severity of variceal bleed. Based on the data, it was unanimously agreed that acute variceal bleeding is not an acute hepatic insult unless in the patients where it produces jaundice and coagulopathy fulfilling the criteria of ACLF.

(D) *Acute HVOTO or PVT* presenting as ACLF is a novel concept. The disease burden, clinical picture, prognosis and treatment strategies of BCS or PVT presenting as ACLF are largely unknown. The thrombophilic disorders in those with ACLF have not been evaluated but are unlikely to be different from those without ACLF. The reduction of hepatic blood flow due to acute PVT may lead to ischemic liver injury [51]. The diagnosis of acute-on-chronic BCS in the study by Langlet et al. was based on the presence of both acute and chronic features, clinically and/or radiologically [52]. However, the entity of ACLF was not described at that time and it is unclear if any of these patients would have fulfilled the criteria of ACLF. However, it was reported that these patients had worse outcome as compared to other patients with Budd–Chiari syndrome. Evaluation of thrombophilic disorders in patients with PVT or BCS and ACLF should be similar to those without ACLF. There is no evidence currently to suggest that non-cirrhotic portal fibrosis or EHPVO may present as ACLF.

The issue of other non-hepatotropic insults which have been considered in other studies such as surgery, trauma, insertion of transjugular intrahepatic portosystemic shunt, trans-arterial chemoembolization or radiofrequency ablation for hepatocellular carcinoma was discussed in detail. While there is an indirect connection with each of these, it was debated that a patient who already has cirrhosis with HCC or a cirrhotic who undergoes surgery, separate risk scores are already in practice and being utilized. The likely potential for hepatic decompensation would vary depending on the nature of intervention and underlying hepatic reserve. It was agreed that non-hepatotropic insults producing direct hepatic insult and ACLF in an otherwise compensated liver disease could be considered as acute hepatic insults. In a proportion of patients in Asia or even in the west, the precise agent(s) leading to acute hepatic insult are not well recognized on routine assessment. In such patients, this should be recorded as such.

**Recommendations**

2.1. ***Defining the acute event in ACLF.***
2.1.1. Infections.
2.1.1. *Hepatotropic infections*.
2.1.1.1. Hepatotropic viral infections: In this group, reactivation of Hepatitis B virus (HBV) infection and super-infection with hepatitis virus are the major causes of acute insults for precipitating ACLF (1, A).
2.1.1.2 Hepatotropic non-viral infections: These include bacterial, parasitic, and fungal infections precipitating liver failure and ACLF (2, C).
2.1.2. *Non-hepatotropic infections*.
2.1.2.1. Bacterial infection is unlikely to be the precipitant in individuals with a definite hepatotropic acute insult (2, B).
2.1.2.2. Bacterial infections, if they primarily precipitate hepatic failure, and present as ACLF, may be considered as a precipitant of ACLF, but data at present are inadequate to demonstrate that infection *per se* could lead to jaundice and liver failure (2, C). Drug-induced liver injury (Drugs, CAM & HDS)—with or without cirrhosis.
2.1.2.1. Drug-induced ACLF (ACLF-D) is a distinct entity than DILI [1, A].
2.1.2.2. Diagnosis of ACLF-D is challenging as liver disease-related fluctuations in the liver function tests may be part of the natural history of the disease and may confound the diagnosis. Further, cirrhotic patients may not show marked derangements in transaminases [1, B].
2.1.2.3. Those who develop ACLF-D are likely to have severe consequences including decompensation and death (1, B).
2.1.2.4. Drugs responsible as acute insults, triggering ACLF-D in cirrhosis patients include anti-tubercular drugs, Complimentary and alternative medications, antiretroviral drugs and Methotrexate (1, B). More evidence is needed for drugs like azithromycin, azole antifungals and antimicrobials in cirrhotics (2, B).
2.1.2.5. Risk of liver injury is proportional to the number of hepatotoxic drugs in anti-TB regimen (2, C).
2.1.3. *Autoimmune liver disease—distinction in presentation as ACLF and ALF.*
2.1.3.1. ACLF-AIH frequently presents as seronegative for autoantibodies or normal IgG levels [B2]. Seronegative AIH cases might be overlooked without histology [1, B].
2.1.3.2. Diagnosis of ACLF-AIH requires liver biopsy (transjugular route preferred). Biopsy is more helpful in patients where etiology is not evident; antibodies and IgG are negative but there is a high index of suspicion (like extrahepatic features of autoimmunity/family history of autoimmunity or autoimmune diseases like vitiligo, thyroiditis) [1, B].
2.1.3.3. Frequency/degree of fibrosis may define chronicity (ACLF or ALF), but fibrosis may progress in a few weeks from F0 to F1–2 [2, C].
2.1.3.4. Corticosteroid therapy should be considered for a select group of patients presenting with ACLF-AIH [2, B].
2.1.4. *Acute variceal bleed.*
2.1.4.1. The frequency of acute variceal bleed (AVB) increases with severity of cirrhosis [2, B}.
2.1.4.2. AVB in compensated cirrhosis (Child A) leads to the development of ACLF in less than 5% cases [2, B].
2.1.4.3. Mortality in compensated cirrhosis increases with the development of ACLF in 90 day follow-up post-variceal bleed [2, B].
2.1.4.4. Incidence of post-EVL ulcers in ACLF is higher than that in cirrhosis [1, C].
2.1.4.5. Though infrequent, AVB can lead to ACLF in small proportion of Child A patients. Further studies are required in patients with Child B [2, C].
2.1.5. *Vascular liver diseases (PVT, HVOTO).*
2.1.5.1. In patients with cirrhosis, development of acute occlusive portal vein thrombosis (PVT) may precipitate ACLF in a small sub-group (2, C).
2.1.5.2. In patients with cirrhosis or Budd–Chiari syndrome, development of acute hepatic vein thrombosis (PVT) may precipitate ACLF (2, C).
2.1.5.3. Patients with Budd–Chiari syndrome (BCS) may infrequently present with or develop ACLF (2, C).
2.1.5.4. Evaluation of thrombophilic disorders in patients with PVT or BCS and ACLF should be similar to those without ACLF (2, C).
2.1.5.5. There is no evidence currently to suggest that non-cirrhotic portal fibrosis or EHPVO may present as ACLF (2, C).
2.1.5.6. No data are available about the natural history or outcome of patients with PVT or BCS presenting with ACLF and no recommendations can be made for management of patients with vascular liver diseases and ACLF (2, C).

#### Defining the underlying chronic liver disease

Two aspects were carefully analyzed, what constitutes chronic liver disease, cirrhosis alone or non-cirrhotic chronic liver diseases and the etiology of the chronic liver disease.

The degree of hepatic fibrosis and the functional hepatocellular mass remains heterogeneous in patients with the chronic hepatitis [53, 54]. Even in patients with stage IV fibrosis, critical mass varies according to the parenchymal reserves. Modified Laennec Scoring System divides stage IV further, according to the thickness of septa into three, ending up in six stages altogether [55, 56]. Moreover, ACLF is not equivalent to the acute decompensation of cirrhosis, which has protean manifestations. Majority of the ACLF patients present with liver failure without any previous assessment of liver disease. It is not possible to distinguish accurately the natural history of patients with different degree of fibrosis presenting with ACLF at this point in time. The liver with any significant degree of fibrosis, with activated stellate cells, and infiltrated by the inflammatory cells, is expected to respond in a different way to the acute insult compared to the liver without inflammatory infiltrate [57].

NAFLD is the leading cause of donor rejection in liver transplantation [58]. Experience from liver transplantation centers shows that steatosis greater than 30% in the donor liver is associated with a higher risk of primary non-function and graft initial poor function as compared to grafts with no or less than 30% steatosis [59]. Patients with metabolic syndrome and fatty liver, diabetics, male patients of age greater than 45–50 years, and patients with obesity and dyslipidemia have increased risk of fibrosis [60]. While cirrhosis could be a late event, a large proportion of patients may have stage 2 or 3 fibrosis. Hence, NASH is indeed an important cause of chronic liver disease [61]. Furthermore, in the East, a large proportion of patients do have reactivation of chronic hepatitis B. In these patients, while liver failure and ACLF-like presentation do develop, cirrhosis is not necessarily present. The AARC data based on the liver biopsy studies corroborated the facts that a fair proportion of patients with ACLF do not have full-fledged cirrhosis, but still carry a poor prognosis, with mortality above 33% at 4 weeks. Based on the current data set, and published literature and the validity of the 2009 consensus on including the non-cirrhotic chronic liver disease were reaffirmed.

Accurate and reliable assessment of underlying CLD in the setting of ACLF is important for the subsequent management and need for liver transplant in these patients. Diagnosis of chronic liver disease in the setting of ACLF is made by history, physical examination, and previously available or recent laboratory, endoscopic or radiological investigations [62]. Ultrasound and CT abdomen may pick up CLD. However, to assess the degree of fibrosis in an un-shrunken liver would require other radiological modalities. The current non-invasive tests cannot clearly diagnose the presence of chronic liver disease in the presence of inflammation and liver failure. Hence, liver biopsy through the transjugular route or occasionally through laparoscopy remains an important tool to confirm the stage of fibrosis and presence of cirrhotic or non-cirrhotic liver disease.

A liver biopsy through the transjugular route may be of help when the presence of already underlying CLD and the cause of liver disease are not clear. The liver biopsy may highlight the etiology, stage of fibrosis, prognosis and outcome in patients with ACLF [63]. In addition, transjugular access directly into the hepatic vein allows the hepatic venous pressure gradient to be measured (HVPG). There is a risk of bleeding leading to hemobilia, hemoperitoneum, and hepatic hematoma in the setting of the deranged clotting profile [64]. The need of liver biopsy in ACLF should, therefore, be individualized. Standardization of liver biopsy assessment would help a uniform approach to the diagnosis and treatment of CLD and the acute insult.

There is a need to have reliable non-invasive tools to assess the severity of fibrosis in a previously undiagnosed CLD. Ultrasound and CT abdomen may pick up CLD. However, to assess the degree of fibrosis in an unshrunken liver would require other radiological modalities. Transient elastography is a good modality to detect hepatic fibrosis [65]. However, the liver tissue stiffness may also increase with hepatitis, steatosis, and inflammation present in the ACLF setting [66].

The second issue was about the etiology of chronic liver disease and cirrhosis in the Asian pacific region. The experts reviewed the data from the AARC database and the etiologic profile of cirrhosis in ACLF was found to be similar to etiology of cirrhosis in general in the respective countries [28, 67, 68]. With the rising incidence of obesity and NAFLD, a proportion of burnt-out NASH presenting as cryptogenic cirrhosis also increases [69–71]. Viral serology and nucleic acid testing are required to identify viral etiology. Specialized tests to diagnose metabolic and autoimmune diseases are needed as well. The presence of stigmata of liver disease on clinical examination, low platelets, evidence of synthetic dysfunction in previous reports, and altered AST/ALT ratio in previous reports should prompt the diagnosis of the presence of CLD [72, 73].

**Recommendations**

2.2. ***Defining the underlying CLD:***
2.2.1. Cirrhotic and non-cirrhotic chronic liver diseases qualify as chronic liver diseases (1, A).
2.2.2. The common underlying chronic liver diseases include alcohol, hepatitis B, hepatitis C, NAFLD-related chronic liver disease or cirrhosis of the liver (1, A).
2.2.3. Chronic hepatitis and/or significant fibrosis without cirrhosis should be taken as a chronic liver disease, if such a patient presents as ACLF (1, B).
2.2.4. NAFLD-related chronic hepatic injury; NASH, if associated with significant fibrosis, should be taken as a chronic liver disease in ACLF (1, B).
2.2.5. Patients with known previous decompensation with jaundice, HE, and ascites should be excluded (1, C).
2.2.6. Diagnosis of chronic liver disease and cirrhosis in the setting of ACLF is made by history, physical examination, laboratory, endoscopic or radiological investigations (1, A).
2.2.7. A liver biopsy through the transjugular route may be helpful when the presence of underlying chronic liver disease and/or the cause of chronic liver disease and/or the acute insult is not clear (1, B).

#### Impact of comorbidities and obesity

Comorbidities also influence the outcome of ACLF as far as the disease and outcome are concerned. The presence of co-morbidities like obesity, sarcopenia and other metabolic risk factors like diabetes mellitus, hypertension and dyslipidemia have a bearing on the outcome of patients with cirrhosis [74]. However, there is a sparse literature on the effect of obesity, sarcopenia and other metabolic risk factors on the severity and outcome of patients with acute-on-chronic liver failure (ACLF). In a recent analysis of the AARC database, the prevalence of metabolic risk factors and its impact on the severity and outcome were analyzed in patients with alcohol-related ACLF as per the APASL definition [75]. In a recent report, of the 1028 patients from AARC database, 15% patients had history of overweight or obesity, 14% of T2DM, 7% of HT and 15% of dyslipidemia. Patients with metabolic traits compared with control group, had more severe disease; those with overweight or obesity had significantly higher MELD score and those with dyslipidemia had higher AARC score. None of the other metabolic risk factors either alone or in combination had any impact on the severity of ACLF. The presence of overweight or obesity was also significantly associated with increased day 30 mortality while none of the other metabolic risk factors, alone or in combination were associated with day 30 or 90 mortality [75]. In addition to above, alcohol intake and subsequent chronic liver disease with or without cirrhosis is another co-morbid condition.

**Recommendations**

2.3. ***Impact of comorbidities and obesity***.
2.3.1. The presence of overweight or obesity and dyslipidemia increases the severity of liver disease in ACLF patients (1, B).
2.3.2. The presence of overweight or obesity increases the short-term (30 day) mortality in patients with ACLF (1, B).
2.3.3. There is need to compare the development and natural history of ACLF in patients with NASH versus NASH cirrhosis (2, C).

#### Changing trends for the etiology of acute insult and chronic injury

The epidemiology of acute insult has changed significantly in the past 5 years. In the recent AARC data, alcohol has now emerged as the most common etiology for acute insult (49%) as well as for underlying chronic liver disease in contrast to previuos data of HBV predominance. DILI and autoimmune etiologies have shown increasing trend; however, HAV/HEV had decreasing trend. HBV infection-induced ACLF as well as HAV/HEV-induced ACLF is now showing a decreasing trend over time, whereas alcohol and herbs, drugs and supplements (HDS)-induced ACLF show an increasing trend. The unknown causes for acute insult and chronic injury constitute only 5–15% cases of ACLF in the East in contrast to the West, where these are seen in about 40% of ACLF patients.

**Recommendations**

2.4. ***Changing trends for the etiology of acute insult and chronic injury.***
2.4.1. Alcohol is now the commonest etiology for acute hepatic insult as well as for the underlying chronic liver disease in the Asian continent [2, B].
2.4.2. DILI and autoimmune etiologies have shown increasing trend [2, B].
2.4.3. HBV infection–reactivation of hepatitis B-induced ACLF as well as acute HAV/HEV-induced ACLF shows a decreasing trend over time in certain regions, whereas alcohol and herbs, drugs and supplements (HDS)-induced ACLF show an increasing trend (1, A).
2.4.4. The unknown causes for acute insult and chronic injury constitute only 5–15% cases of ACLF in the East in contrast to the West, where these are seen in about 40% of ACLF patients (1, A).

#### ACLF is distinct from acute decompensation (AD): differentiating AD and ACLF

The two disease entities look similar and are often misunderstood. The experts reviewed the literature and presented their data. The data from the AARC database were presented. The discussion revolved around the following main issues:

*Acute decompensation* occurs in a cirrhotic with or without prior decompensation and is often associated with a precipitant [6]. The presentation of AD is either hepatic (jaundice, ascites, HE) or extrahepatic (variceal bleed, acute kidney injury or sepsis), and time period is up to 3 months [11]. The level of jaundice could be well below 5 mg/dl, below the cutoff generally taken for liver failure. The precipitant for AD can be hepatic (48%) or non-hepatic (46%). Ascites/HE/AVB may precede jaundice. There could be several combinations in the acute decompensation; such as jaundice with or without ascites, HE alone or with ascites with or without jaundice, HE variceal bleed alone or with ascites, sepsis with jaundice or alone, etc. Each of these entities is in themselves, a well-defined complication, and has been extensively studied in patients with cirrhosis. Moreover, AD can be the index event or it could be a repeat event in patients with prior decompensation. Hence, there are multitudes of combinations possible in a patient presenting with AD. After due deliberations, it was unanimously felt that AD should be considered as a recordable time point, an unfavorable event, in the natural history of cirrhosis rather than a syndrome by itself. The precise type of acute presentation of the patient should be recorded and the patient should be accordingly monitored and managed.

The overall mortality in patients with AD at 1 and 3 months was 23% and 29%, respectively, much lower than when patients develop ACLF. The probability of reversal, progression to end-stage liver disease and need for a liver transplantation would depend on the presentation of the AD such as variceal bleed or ascites. Role of bridging therapy and emerging therapies in AD is largely unknown.

Acute-on-chronic liver failure (ACLF) is a syndrome of hepatic decompensation (jaundice, coagulopathy, ascites and/or HE), where the insult is only hepatic and leads to liver failure in a period of 4 weeks [2]. Jaundice and coagulopathy precede development of ascites. Non-hepatic organ failure, i.e., AKI, sepsis, AVB develops after the ACLF syndrome or less commonly, with the onset, depending on the severity of liver failure. The presentation is index, occurring in a patient of chronic liver disease with or without underlying cirrhosis of the liver. The hepatic reserve may show recovery leading to complete reversal of the syndrome as well as regression of fibrosis and portal hypertension. The long-term survival, i.e., after 24 months of index presentation with ACLF is better than the AD cohort [HR: 1.94 (1.17–2.21), *p* < 0.01] [76]. The progression of disease and onset of multi-organ failure are faster in the non-salvageable cohort with a high 3-month mortality of 54% [77].

Development of ascites represents a state of acute portal hypertension in ACLF patients. This rapid rise in portal pressure is a result of severe hepatic inflammation and ongoing liver failure. Highly activated stellate cell population, cytokine storm and ongoing hepatic parenchymal necrosis perpetuate the portal hypertension syndrome. The use of non-selective beta-blockers has been shown to be effective in reducing the mortality and risk of variceal bleed in ACLF patients.

ACLF is a unique disease entity and is distinct from acute decompensation by considering only those patients who have one type of AD and in a specified time frame of 28 days; this includes patients who develop after a hepatic insult, jaundice and coagulopathy followed by development of acute portal hypertension in the form of ascites and or HE (Table 3).

**Table 3:** Differentiating ACLF from acute decompensation

Parameter(s)	Acute-on-chronic liver failure (ACLF)	Acute decompensation (AD)
Presentation	Hepatic insult Index	Hepatic or non-hepatic Can be index or subsequent
Identifiable precipitant	In up-to 95% cases	In up to 70% cases
Time from insult to presentation	Within 4 weeks	Up to 12 weeks
Underlying cirrhosis	May or may not be present	Always present
Prior decompensation	No	With or without Prior Decompensation
Mortality at 1 and 3 months	33–51%	23–29%
Reversal or recovery	In half of cases	Uncommon

**Recommendations**

2.5. **ACLF is distinct from acute decompensation (AD): differentiating AD and ACLF.**
2.5.1. ***Natural history and outcome of ACLF.***
2.5.1.1. The main etiologies for ACLF are alcohol-related injury, viral hepatitis, drug-induced liver injury, and autoimmune liver disease. In the Asian Pacific region, in only about 5–10% of the cases, the acute insult is unidentifiable [1, A].
2.5.1.2. Age and the presence of cirrhosis are independent risk factors for mortality in ACLF (1, B).
2.5.1.3. Portal hypertension with an HVPG greater than 18 mmHg and/or variceal bleeding, presence of complications including ascites, SBP and encephalopathy are independent predictors for mortality (2, B).
2.5.1.4. Starting NSBBs is safe in ACLF and its use is associated with improved short-term survival (2, B).
2.5.1.5. Appropriate management has key impact on the outcomes of ACLF, early (within 2 weeks) anti-HBV treatment for HBV-ACLF, corticosteroid therapy for alcoholic ACLF and AIH-ACLF are worthwhile options (1, B).

2.5.2. ***Natural history and outcome of acute decompensation.***
2.5.2.1. Acute decompensation (AD) is currently defined as acute occurrence of decompensating events (ascites, HE, jaundice, variceal bleed or bacterial infection) in a patient with CLD (1, B).
  2.5.2.2. Patients with AD who have or progress to develop extrahepatic organ failure have high short-term mortality (1, A).
  2.5.2.3. Early evaluation of potential predictors and precipitating agents can help in managing these patients (1, B).

2.5.3. ***Acute decompensation—differentiating from ACLF for clarity in definition.***
2.5.3.1. AD develops in a patient with chronic liver disease/cirrhosis, with or without prior decompensation, and is often associated with an identifiable precipitant and develops in less than 3 months [2, A].
2.5.3.2. Any decompensation preceding jaundice strongly favors AD [1, B].
2.5.3.3. Absence of repeated episodes of decompensation differentiates ACLF as a unique syndrome [2, A]. This has implication on the management decisions and prognostication, including reversibility of the syndrome.
2.5.3.4. Long-term survival, reversal and/or recovery of hepatic reserve has been documented with ACLF [2, A].
2.5.4.5. The differentiating features between different presentations of AD and the ACLF need to be studied carefully by expanding the AARC database [3, C].

#### Role of Liver histology in ACLF

Since the previous consensus statement, new data and insights into the liver histopathology have become available. The main questions that were addressed in the current consensus meeting were: (1) Is liver biopsy feasible and safe in ACLF, (2) Can liver biopsy help to differentiate ACLF from ALF and chronic liver disease, (3) Are there any histological predictors of outcome in ACLF, such as need for liver transplantation or mortality and (4) Are there any differences in regenerative response in sequential biopsies of survivors and non-survivors?

Percutaneous liver biopsy is generally not feasible in patients with ACLF due to coagulopathy and ascites. Transjugular liver biopsy (TJLB), on the other hand, is considered relatively safe and can help assess stage of fibrosis and severity of hepatic injury. For example, severity of alcoholic hepatitis in alcoholic liver disease-related ACLF could be assessed only by liver biopsy [63]. It can provide clues to the underlying acute insult as in Wilson’s disease, malignancy, autoimmune hepatitis, DILI and NASH [78, 79].

Mini-laparoscopic liver biopsy is another alternative for getting liver biopsy in patients of advanced cirrhosis with acceptable bleeding risk. More data needed on this modality and comparison with TJLB are lacking at present. However it can be considered in areas with poor access to TJLB and biopsy is definitely needed for the decision- making [80].

Differentiating ALF and chronic hepatitis with flare is based on findings of fibrous bands (spurs and bridges) and ductular proliferation. Features of cholestasis and bile duct proliferation are more common in patients with acute injury (classical features of acute hepatitis along with cellular and ductular cholestasis are indicative of acute injury). Differentiation between cirrhosis with acute deterioration and compensated cirrhosis is based on the presence of necrosis and features of acute hepatitis in the former group of patients [63]. It was proposed that the diagnostic stains for fibrosis and necrosis be mentioned. It was also proposed that connective tissue stains (especially Shikata’s orcein) should be done in all such cases for differentiating necrosis from fibrosis.

Liver histopathology could be very useful in prognosticating the outcome in a patient with ACLF [63, 81]. The extent of necrosis, liver damage and fibrosis is helpful. The presence of ductular bilirubinostasis on liver biopsy defined as the presence of bile plugs in dilated ductules at the interface between the portal tract and parenchyma predicted a poor outcome and a high potential for development of infections in ACLF. While ballooning was helpful, suggestive of regenerating potential, the presence of eosinophilic degeneration of hepatocytes was not a favorable feature. Standardization of liver biopsy assessment is essential for a uniform approach to the diagnosis and treatment of CLD and acute insult.

Liver regeneration is considered to play an important role in ACLF as prognosis can be improved if the critical threshold of functional liver cell mass is regained. Decompensated cirrhosis is considered irreversible owing to loss of regeneration potential. Liver histology can provide morphological evidence supporting these concepts and for assessing regenerative potential and prognosis [82]. In this report, immuno-histochemical study of two levels of regenerative responses in liver failure revealed that proliferating hepatocytes were significantly more in ALF in comparison to ACLF (*p *< 0.001) and CLD (*p *< 0.001).

There is significant relationship between HSCs and presence of HPCs, indicating a possible dynamic role of HSCs in liver regeneration and pathobiology of ACLF [83]. Liver biopsy is an important mode of understanding and validating the results of clinical trials exploring various therapeutic options for, e.g., mobilization of bone marrow-derived stem cells with granulocyte colony-stimulating factor (GCSF) [84].

***Recommendations***

2.6. **Role of liver histology in ACLF.**
2.6.1. A liver biopsy through the transjugular route is helpful to diagnose/confirm the cause of acute injury [1, B].
  2.6.2. Liver biopsy is helpful in patients where the presence and stage of underlying chronic liver disease and/or the cause of chronic liver disease are not clear (2, A). Biopsy can help identify unsuspected/multiple etiologies, i.e., primary and concomitant etiologies.
2.6.3. The need of liver biopsy in ACLF should be individualized, especially in alcoholic hepatitis, severe autoimmune hepatitis, and flare of Wilson disease (2, A).
2.6.4. Liver biopsy indicates the stage of fibrosis and is helpful in the prognosis and outcome in patients with ACLF (B 1). It can help in distinguishing ACLF from decompensated cirrhosis [1, B].
2.6.5. Certain histologic parameters are predictors of prognosis of ACLF, like ductular bilirubinostasis, eosinophilic degeneration, and parenchymal extinction (1, B).
2.6.6. It can help in distinguishing ACLF from decompensated cirrhosis [1, B].
2.6.7. Standardization of liver biopsy assessment is essential for a uniform approach to the diagnosis and treatment for CLD and acute insult (2, C).
2.6.8. Noninvasive tools to measure liver stiffness and biomarkers may help in identifying patients with advanced fibrosis. Studies are needed to validate the performance of these tests in the setting of ACLF (2, C).

### Defining the liver failure in ACLF

Acute liver failure is generally defined as development of HE within 4 weeks of onset of jaundice [1, 2]. Since the basic premise in ACLF is to identify patients with chronic liver disease or cirrhosis presenting as acute liver failure, the time frame for liver failure was kept as 4 weeks [5].

The clinical presentations in ACLF is varied and depends upon the severity of acute insult and degree of underlying chronic liver disease. In the published reports, patients included as having ACLF had severe jaundice associated with organ failure manifested as either HE or hepatorenal syndrome (HRS) [1, 2].

Defining the liver failure in ACLF, therefore, required a detailed consideration of all the existing liver failure scores and the criteria defining liver failure in the organ failure scores such as SOFA and APACHE II. The two main variables are bilirubin and coagulopathy.

*Serum bilirubin* analysis of the AARC data revealed that patients with a bilirubin between 5 and 10 mg/dl also had substantial mortality ranging around 38%. The data for patients below this level were, however, not collected as per the initial definition, but is likely to yield mortality rates much below 33%. On the other hand, in the CANONIC study, the level of bilirubin for hepatic failure was taken as 12 mg/dl so as to determine 15% mortality at 28 days. If these criteria were applied to the ACLF patients in the Asian region, a much higher mortality was observed in this cohort. After detailed discussion, the original value of > 5 mg/dl was accepted as the cutoff for bilirubin for defining liver failure [1, 2]. This was reiterated to be inclusive of less severe group of patients and enabling a complete spectrum of patients, including those who have a potential for recovery [2].

*Coagulopathy* the presence and degree of coagulopathy as a marker for liver failure were re-evaluated. Coagulopathy is an important hallmark of severe hepatic dysfunction [59, 60]. Patients with ACLF have complex hemostatic defects leading to a delicate, unstable balance between bleeding and thrombosis [85].

Development of clinical ascites and/or encephalopathy has been conventionally taken as evidence of hepatic failure [1, 27]. Ascites and HE were not seen in all the patients and, therefore, presence of either of them was accepted for the definition of ACLF. In the AARC data, ascites was present in 91% and HE in about 45% of the patients at presentation.

The data were further analyzed to see if a shorter interval of 2 weeks instead of 4 weeks is a better cutoff for predicting mortality in patients with underlying cirrhosis who developed jaundice followed by ascites. The analysis of the AARC data showed that in patients who developed ascites within 2 weeks against those after 2 weeks of onset of jaundice, though had a slightly more severe course, the differences in mortality were not significant.

*Grade of liver failure for ACLF* like in many conditions in medicine, such as the NYHA classification for heart failure [86] severity of a disease or variable can be defined to predict the outcome of the disease. Using the four variables, bilirubin, INR, ascites and HE, a simple scoring system may be helpful for making treatment strategies.

The basic premise for defining a syndrome is to identify a group of patients, who have a distinct presentation, course and outcome. A prospective study using AARC database with 1402 patients from several centers across Asia included a large derivation cohort of 480 patients to develop a dynamic prognostic model, which was validated in subsequently enrolled 922 patients to predict outcomes including mortality [3]. The results bring forth a simple ‘liver failure grading system’ for patients with chronic liver disease based on variables, namely serum bilirubin, INR, grade of HE, serum lactate and serum creatinine [43, 87–89]. Serum lactate levels are elevated in relation to degree of hepatocellular injury, inflammation and degree of tissue perfusion.

The analysis resulted in a simple ‘liver failure grading system’ based on 5 variables; namely, serum bilirubin, INR, serum lactate, serum creatinine and grade of HE. There is no score dedicated to liver failure in cirrhotic patients, commonly recognized as a distinct entity, ACLF. The grading system, i.e., Grade I for a score of 5–7, Grade II for 8–10 and Grade III for 11–15 with 28-day mortality of 12.7, 44.5 and 85.9%, respectively, was developed. The grades of liver failure showed a potentially recoverable group (Gr. I), a group that needs special monitoring (Gr. II) and a group that demands immediate interventions for improved outcome (Gr. III). The AARC model was found to be better than existing models for ACLF with an excellent predictability, i.e., in AUROC of 0.80 (derivation cohort) and 0.78 (validation cohort). It is even more robust than recently reported models [3] where the AUROC is below 0.80. The AARC-ACLF score is dynamic in nature. It could predict day 7 (score of 9 or below) and day 28 survival at presentation (score of 9 or below). For a baseline score of ≥ 10, with each one unit increase, the day 7 mortality increased sharply compared to the patients who presented with a score of < 10 (20% vs. 4%). The score also predicted well the day 28 and day 90 survival. Thus, the AARC score provides a physician a window to decide early and explore definitive therapies including liver transplantation. Shift from grade I to III liver failure at day 4 and day 7 increases the mortality significantly. At the same time, the persistence of grade I or II liver failure till day 7 predicted improved survival, while persistence in grade III failure was uniformly severe and warranted early consideration for transplant [3].

**Recommendations**

3.0. ***Defining the liver failure in ACLF:***
3.1. Jaundice (serum bilirubin > 5 mg/dL [> 85 lmol/L]) and coagulopathy (INR > 1.5 or prothrombin activity < 40%) are mandatory parameters to assess liver failure (1, A).
3.2. Ascites and/or encephalopathy as determined by physical examination also reliably reflect significant hepatic functional impairment (1, A).
  3.3. Liver failure score (AARC score) which includes total bilirubin, INR, grade of HE, plasma lactate and serum creatinine reliably predicts the disease severity and outcome (1, A).
3.4. Grading of liver failure as per AARC score I (5–7), II (8–10), III (11–15) effectively prognosticates and guides the therapy [1, B].
3.5. The assessment of coagulation system by global coagulation methods (viscoelastic technique/thrombin generation test) may be considered as a useful tool for assessing coagulation anomalies in ACLF patients (2, B).

### Sepsis in ACLF

Sepsis is a syndrome of systemic inflammatory response of the host to an identifiable infection. The systemic inflammatory response syndrome (SIRS) is defined by the presence of at least two of the following criteria: (1) altered temperature, (2) elevated respiratory rate or hyperventilation, (3) tachycardia, and (4) altered white blood cell count (high, low, or immature forms) [67]. Sepsis is the most common cause of mortality in most intensive care units (ICUs) [90–92].

**Fig. 3:**
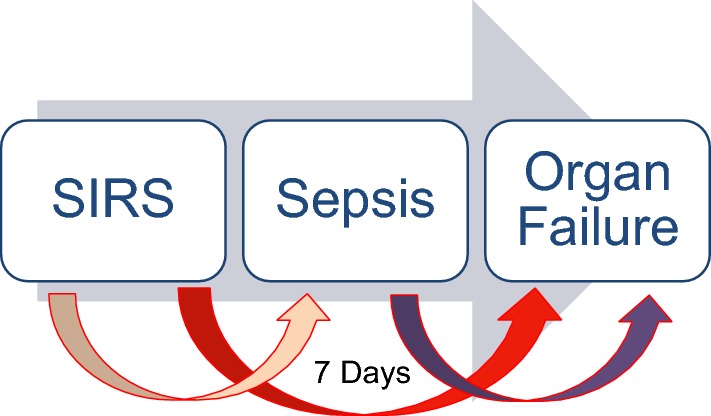
Golden window in ACLF. ACLF is the state of acute inflammatory response with cytokine burst. The SIRS is a response to this inflammation and subsequent resolution of inflammation and recovery or persistence of inflammation (leading to Compensatory Anti-inflammatory Inflammatory Response Syndrome-CARS) and sepsis. Patients of ACLF in a period of 7 days develop SIRS (which can be infective or sterile) but both the things lead to complications and sepsis develops subsequently. This time period is the therapeutic Golden Window. SIRS needs consideration for organ support, antibiotics for occult sepsis and prioritization for definitive therapy, i.e., liver transplant

Due to the hyperdynamic circulation and complications of portal hypertension, the currently accepted clinical definition of SIRS and sepsis may not be entirely applicable to patients with cirrhosis or ACLF. Hence, a high index of suspicion is required for making a clinical diagnosis of sepsis in these patients. Liver failure initiates and predicts the development of SIRS. New onset SIRS in the first week is an important determinant of early sepsis, organ failure, and survival (Fig. 3). Prompt interventions in this ‘golden window’ before development of sepsis may improve the outcome of ACLF [78].

Consideration of sepsis as an acute insult in the absence of liver failure is confusing and with limited scientific basis. Sepsis is a consequence rather than the cause for development of ACLF. The APASL definition does not include sepsis as a primary cause for liver failure, but in the Western definition, sepsis is considered as the most common precipitant. The inclusion of sepsis in the definition is likely to be associated with concomitant multi-organ involvement, poor prognosis and would be unlikely to provide a targeted therapy or a definitive therapy such as liver transplant.

SIRS is the inflammatory response to the damaged organ in the host. It could be a result of sterile inflammation or an occult infection [93–95]. In fact, in 60% of patients fulfilling the SIRS criteria, infection could not be detected [78]. This highlights the limitations of the current techniques available to detect infections or may be because of use of prophylactic antibiotics the detection of organisms becomes difficult. Prevention of development of SIRS or its progression from SIRS to sepsis by immune modulation in the ‘Golden Window’ period could decrease the incidence of organ failure and improve survival [78]. ‘Golden window’ is a short period of about 1 week before the onset of sepsis and development of extrahepatic organ failure in a patient with ACLF. Therapeutic interventions during this period are likely to prevent organ failure and provide a potential opportunity for ameliorating or reversing the hepatic injury and failure [78].

The current paradigm regarding the host immune response to sepsis is debated and is a matter of great interest in clinical trials as well as basic science. Two theories have been proposed to describe the host response to sepsis. According to the most accepted theory, both pro-inflammatory and anti-inflammatory responses occur early and simultaneously in sepsis although the net initial effect of these competing processes is typically manifested by an early, dominant, hyperinflammatory phase characterized by shock, fever and hypermetabolism [96, 97]. Subsequently, this initial hyperinflammatory phase evolves over several days into a more protracted immunosuppressive phase [98]. The robustness of the hyperinflammatory phase depends on numerous factors, including pre-existing co-morbidities, nutritional status, microorganism load and virulence factors [99, 100].

According to the second theory, there is rapid and sustained upregulation of genes that regulate the innate immune response and the simultaneous downregulation of genes that regulate the adaptive immune response. There is protracted, unabated inflammation driven by the innate immune system with resultant organ dysfunction and failure [101].

Whether sepsis is the cause or a result of liver failure was debated at length. The fact that patients who presented with no SIRS or subsequently developed SIRS or sepsis over a period of 1–2 weeks indicates that infection and sepsis develop after liver failure and unabated inflammation leads to immune paresis and provides an opportunity for infections and sepsis to occur. Non-hepatic infections are also common in patients with ACLF [101, 102]. Neutrophil dysfunction and immune paralysis due to reduced HLA-DR expression have been shown to rapidly develop in ACLF patients [102–104]. The frequency of intrahepatic myeloid and plasmacytoid dendritic cells is reduced with increased interferon gamma producing CD8 T cells in patients with ACLF. Decreased frequency of DCs and high IFN-γ levels correlate with poor patient survival [104].

Bacterial infection is present in about 1/3rd of ALCF patients at presentation to a tertiary referral hospital, and this further increases by first week [78]. The AARC data showed that the patients presenting with sepsis, at baseline or who developed new sepsis at day 4, have high mortality. Bacterial infection (BI) predicts development of organ failure in ACLF. Organ dysfunction and organ failure are significantly higher in infected cohort with high short-term mortality. The acute phase proteins, such as C-reactive protein and procalcitonin, were proven to be reliable biomarkers for bacterial infection. The most frequent infections are SBP, pneumonia, UTI, and bacteremia. Second infection (2nd hit) is associated with poor outcome in patients with ACLF. Hospitalized patients with ACLF should be monitored closely for the presence of infections to enable early diagnosis and treatment. Routine examination of blood and body fluids is recommended. Patients who respond to treatment for bacterial infection have significantly reduced mortality. Patients who respond to treatment for bacterial infection have significantly reduced mortality. As soon as bacterial infection is diagnosed or suspected, broad spectrum antimicrobial agents or combined use of antibiotics are preferred, thereafter the therapy is adjusted according to the results of the sensitivity test. Empirical antibiotic therapy should be based on environment, local resistance profiles, severity and type of infection. To optimize the empirical antibiotic treatment, it is quite important to distinguish among community acquired, health care associated and nosocomial infections.

Invasive fungal infection is not uncommon in ACLF patients. However, data are minimal. The diagnosis of invasive fungal infection can be proven, probable or possible, depends on mycological evidence, and clinical evidence. Invasive pulmonary aspergillosis (IPA) is increasingly recognized as a cause of morbidity and mortality in patients with ACLF. Prophylaxis of invasive fungal infection may be applicable with echinocandins in selected patients. Prophylaxis with fluconazole followed by echinocandins needs to be evaluated in ACLF patients. Predictors of poor progression (risk factors) include diabetes, AKI, ICU admission, and admission for bacterial infection, prolonged antibiotic therapy (> 5 days from admission), prior h/o hospitalization. Biomarkers such as galactomanan or B–D Glucan can be used for supporting diagnosis if there is invasive fungal infection in ACLF. Administration of albumin is recommended in patients with SBP for preventing from type-1 HRS and reducing mortality. The role of albumin in preventing or treating other infections in ALCF is not clear.

**Recommendations**

4.0. **Sepsis in ACLF.**
4.1. There is a central role of inflammation and dysbalance of innate and adaptive immune responses in ACLF patient (1, B).
4.2. It is difficult to differentiate SIRS from early sepsis in cirrhosis (2, A).
4.3. Bacterial infection is present in about 1/3rd of ALCF patients at presentation to a referral hospital, and this may increase in the first week [2, B].
4.4 Patients who do not have sepsis have low 28-day mortality [B2]. Patients with accompanying sepsis at baseline or who develop sepsis at day 4 have high mortality [2, B].
4.5 Organ dysfunction and organ failure are significantly higher in infected cohort and this is attended with high short-term mortality [2, B].
4.6 Bacterial infection (BI) is an important factor to predict development of organ failure in ACLF. The most frequent infections are SBP, pneumonia, UTI, and Bacteremia (1, A).
4.7 Second infection (2nd hit) is associated with poor outcome in patients with ACLF [1, B].
4.8 Hospitalized patients with ACLF should be monitored closely for the presence of infections to enable early diagnosis and treatment. Routine examination of blood and body fluids is recommended (1, A).
4.9 The acute phase proteins, such as C-reactive protein and procalcitonin, have been proven to be reliable biomarkers associated with infection and are recommended for screening for the presence of bacterial infections (1, B).
4.10 As soon as bacterial infection is suspected or diagnosed, broad spectrum antimicrobial agents alone or in combination should be started and thereafter, the therapy should be adjusted according to the results of the antibiotic sensitivity test results (1, A).
4.11. Empirical antibiotic therapy should be based on environment, local bacterial resistance profiles, severity and type of infection. To optimize the empirical antibiotic treatment, it is quite important to distinguish among community acquired, health care associated and nosocomial infections (2, A).
4.12. Invasive fungal infection is not uncommon in ACLF patients. These can be proven, probable or possible, depending on mycological and clinical evidences (2, B). Biomarkers such as galactomanan or B-D glucan can be used for supporting the diagnosis (1, B).
4.13. Invasive pulmonary aspergillosis (IPA) is increasingly recognized as a cause of morbidity and mortality in patients with ACLF. Voriconazole plasma concentration monitoring may ensure the safety and efficacy of ACLF patients with Invasive aspergillosis infection (2, C).
4.14. Prophylaxis of invasive fungal infection can be done using echinocandins in selected patients (B2). Prophylaxis with fluconazole followed by echinocandins needs to be evaluated in ACLF patients [1, C].
4.15. Predictors of poor progression (risk factors) of fungal infections in ACLF are the presence of diabetes, AKI, ICU admission, and admission with bacterial infection, prolonged antibiotic (> 5 days pre-admission) and prior hospitalization (2, B).
4.16. The value of qSOFA and Sepsis-3 criteria is unclear to assess severity of infection in patients with ACLF.
4.17. Administration of albumin is recommended in patients with SBP to prevent development of type-1 HRS and reduce mortality (A2). The role of albumin in preventing or treating other infections in ALCF is not clear [2, B].
4.18. Patients who respond to treatment for bacterial infection have significantly reduced mortality [2, B].

### Organ dysfunction and organ failure in ACLF

The concept to differentiate organ dysfunction from organ failure is useful in assessing the degree of organ damage and predicting the probability of disease progression or regression; prioritizing the patient for liver transplantation and the likelihood of futility of care or high probability of death.

The data from AARC database were carefully analyzed with respect to defining organ dysfunction and organ failure for each of the extrahepatic organs.

#### Renal failure

Renal failure in patients of ACLF is considered to be a complex and challenging condition that is associated with an ominous prognosis. Further, kidneys are one of the most frequent extrahepatic organs involved in patients with ACLF. The EASL-CLIF consortium has defined ACLF in which kidney dysfunction is used as a defining condition [11]. Hence, renal dysfunction or failure is universally present in patients with ACLF with or without liver failure, according to the definition by the EASL-CLIF consortium. On the contrary; the APASL definition of ACLF does not incorporate kidney dysfunction in its definition [1, 2]. Studies based on APASL criteria have reported renal dysfunction in 22.8–34% of patients with ACLF and as high as 51% using the more sensitive AKIN criteria [103]. This highlights the fact that a significant number of patients of ACLF based on APASL criteria who do not have renal dysfunction (using even the most sensitive criteria to detect renal involvement) would definitely be missed if renal dysfunction was considered in the definition as per CLIF.

In patients with decompensated cirrhosis, the main abnormality causing renal dysfunction is systemic and splanchnic vasodilatation secondary to portal (or sinusoidal) hypertension which leads to decreased effective arterial blood volume and activation of neurohormonal systems, the rennin–angiotensin aldosterone (RAAS), the sympathetic nervous system and non-osmotic release of antidiuretic hormone, resulting in sodium and water retention [104–106]. The systemic hemodynamic alterations in ACLF are similar to those in patients with decompensated cirrhotics [107], but the pathogenesis of renal dysfunction in ACLF is quite different; a major role is played by SIRS and subsequent sepsis in patients of ACLF but not in those with decompensated cirrhosis [108]. A higher prevalence of structural AKI has been reported for patients with ACLF. In a large cohort study from the AARC database, the concept of PIRO (defined as predisposition, infection/inflammation, response, organ failure) was used for creating a predictive model for understanding the pathophysiology of kidney dysfunction in patients with ACLF [109]. Together, the components of PIRO reflect the role of key factors, which result in development, and progression of AKI in patients with ACLF. The PIRO, which is a composite score, derived from the AARC database accurately predicted in patients with ACLF, highlighting the prognostic significance of kidney dysfunction in these patients. The PIRO score can predict the development of AKI within first 15 days of diagnosis of ACLF and may help in stratification for additional therapeutic interventions [109].

Serum creatinine is used for defining and staging AKI in patients with cirrhosis. However, serum creatinine is influenced by various factors, which affect its production and excretion, as well as those that impact its measurement and, therefore, is unreliable in patients with severe liver dysfunction including ACLF. The AKI criteria seem more relevant as they rely on a dynamic change rather than a static measurement of serum creatinine. A lower value of serum creatinine is more relevant in patients with ACLF. Serum creatinine above 0.7 mg/dl (as derived from the AARC score) has a sensitivity of 78% and specificity of 36% for prediction of 30-day mortality in patients with ACLF. For the diagnosis of kidney failure, the conventional cutoff of 1.5 mg/dl even though had a low sensitivity of 48% but had a specificity of 99.8% for 30-day mortality. Kidney failure (serum creatinine ≥ 1.5 mg/dl) was seen in 22% of ACLF patients at baseline and developed in another 30% within a month. The majority of patients of ACLF developed new episodes of AKI in the first 2 weeks (11%). Further, the KDIGO definition for AKI (incorporating the urine output criteria) has been shown to be more accurate in determining AKI course in critically ill cirrhotics; however, this needs to be evaluated for patients with ACLF [110].

Apart from the severity, the course of AKI was seen to be an important predictor of clinical outcomes. Almost 80% of the patients, who did not present with AKI at baseline or developed within the first 7 days, survived 1 month. However, in patients who presented with AKI, resolution of AKI was associated with a better survival. Progression of AKI was associated with highest mortality of 75%. Development of new AKI as well as non-resolution or persistence of AKI was associated with almost 50% mortality at 1 month in patients with ACLF [109].

Kidney injury biomarkers have been studied in patients with decompensated cirrhosis; however, their utility in diagnosing, staging and understanding the spectrum of AKI including the response to therapies has not been carefully evaluated in patients with ACLF. There is a potential to look at the role of biomarkers of tubular damage, namely *N*-GAL, Kim-1, IL-18 and l-FABP to differentiate functional AKI or HRS from structural AKI, i.e., ATN patients with ACLF [111] as ATN or structural kidney damage may necessitate the need of simultaneous liver–kidney transplant as against liver transplant alone for HRS [112].

Treatment for AKI in ACLF depends on the etiology, severity, complications, and the presence of other organ failure and/or hemodynamic status. Targeting the components of PIRO, i.e., combating systemic inflammation with anti-inflammatory strategies (for instance intravenous albumin, *N*-Acetylcysteine), bilirubin reduction, avoidance of nephrotoxic drugs, aggressive management of circulatory failure and maintaining a high mean arterial pressure may prevent AKI development and progression in patients with ACLF. Albumin intravenous administration has multiple benefits volume correction in addition to its anti-inflammatory and immunomodulatory properties.

Data on use of vasoconstrictors for AKI in ACLF are limited. Terlipressin given as an infusion is superior to noradrenaline in the management of HRS-AKI in patients with ACLF, but needs extra precaution and close monitoring for terlipressin-related adverse effects [111]. The response to vasoconstrictor drug is multifactorial and depends on the severity of AKI, MELD score and AARC grade [113, 114]. Response to vasoconstrictors in AKI in ACLF patients is partial and seen in only in about a third of patients. There is, therefore, quite often a need for renal replacement therapy (RRT). The indications for RRT include severe volume overload, hyperkalemia, hyponatremia and worsening metabolic acidosis not responding to conservative management [115]. Unlike other indications, the threshold for initiating RRT should be relatively low, i.e., when AKI occurs as part of multi-organ failure or in non-oliguric patients, if the daily fluid balance cannot be maintained, Continuous Renal Replacement Therapy [CRRT] should be considered [116]. The mode of dialysis, i.e., hemodialysis (HD), Slow Low Efficient Dialysis (SLED) or CRRT should be chosen based on the hemodynamic status of the patient and the expertise and availability of the equipment [115, 116].

**Recommendations**

5.1. **Renal failure in ACLF.**
5.1.1. *Defining AKI in ACLF.*
5.1.1.1. AKI criteria (defined as an absolute increase in serum creatinine of 0.3 mg/dl within 48 h or by percentage increase in serum creatinine of more than 50% from baseline, which is known, or presumed, to have occurred within the previous 7 days) should be used for the diagnosis of AKI in patients with ACLF [1, A].
5.1.1.2. AKIN criteria should be used for the diagnosis and prognostication of AKI in ACLF patients (1, B).
5.1.1.3. Urine output should be considered to assess the stage and course of AKI in patients with ACLF admitted to the ICU [2, C].
  5.1.1.4. In the absence of baseline serum creatinine, AKI should be diagnosed based on the cutoff value of serum creatinine. A cutoff value of 1.1 mg/dl is a reliable marker of significant renal dysfunction and 1.5 mg/dl of kidney failure in patients with ACLF [1, B].
  5.1.2. *Course of renal failure in ACLF.*
5.1.2.1. AKI is more common and rapidly progressive in patients with ACLF as compared to decompensated cirrhosis and is associated with significantly worse outcome (1, B).
5.1.2.2. Patients with ACLF and AKI have high 30-day mortality. The course of AKI is an important determinant of clinical outcomes [1, A].
5.1.2.3. Progression of AKI has the worst outcome and at the same time resolution is associated with improved survival. Both persistence and new development of AKI are associated with a high short-term mortality in patients with ACLF [1, B].
5.1.2.4. Serum creatinine levels 1.1–1.5 mg/dl or AKIN I are also associated with significant mortality in ACLF patients (2, B).
5.1.3. *Treatment of renal failure in ACLF.*
5.1.3.1. All attempts should be made to prevent development of new AKI in patients with ACLF [1, C].
5.1.3.2. Patients with ACLF should be stratified based on the PIRO (Predisposition, Infection/Inflammation, Response, Organ failure) score for identifying patients at risk of AKI development [1, B].
5.1.3.3. Targeting the components of PIRO, i.e., combating systemic inflammation with anti-inflammatory strategies (for instance intravenous albumin, *N*-Acetylcysteine), bilirubin reduction, avoidance of nephrotoxic drugs, aggressive management of circulatory failure and maintaining a high mean arterial pressure may prevent AKI development and progression in patients with ACLF [2, C].
5.1.3.4. Data on use of vasoconstrictors for AKI in ACLF are limited. Terlipressin given as an infusion is superior to noradrenaline in the management of HRS-AKI in patients with ACLF (B1). Terlipressin use in ACLF should be carefully monitored for adverse effects (1, A).
5.1.3.5. Severity of AKI, MELD score and ACLF grade predicts therapeutic response to terlipressin and albumin in patients with HRS-AKI in ACLF (1, B).
5.1.3.6. New treatments should be explored for patients with AKI-ACLF looking at systemic inflammation as a potential target (2, A).
5.1.3.7. Patients with ACLF with AKI persistence should be considered for additional pharmacologic interventions to prevent AKI progression and enhance AKI resolution [2, C].
5.1.3.8. Initiate RRT emergently when life-threatening changes in volume overload, hyperkalemia, hypernatremia and worsening metabolic acidosis not responding to conservative management (1, C). The threshold for initiating RRT should be lowered when AKI occurs as part of multi-organ failure (1, C), or in non-oliguric patients if the daily fluid balance cannot be maintained.
5.1.3.9. Biomarkers of tubular damage, urine NGAL and IL-18 need to be evaluated for a role in patients with ACLF to determine the need for early RRT or artificial liver support (2, C).

#### Hepatic encephalopathy

The development of HE within 4 weeks of onset of jaundice is part of the criteria for defining acute-on-chronic liver failure (ACLF) [1, 2]. In the recent AARC data, HE was seen to be present in about 40% of the patients. Multiple prospective and retrospective studies had shown that HE in ACLF patients is associated with higher mortality, especially in those with grade III–IV encephalopathy, similar to that of acute liver failure (ALF). The experts proposed and defined cerebral dysfunction and cerebral failure as the presence of grade I and II HE and Grade III and IV HE, respectively. The presence of HE itself increases short-term mortality in ACLF as is the severity, i.e., grade of HE [3].

The pathophysiology of HE is complex, and impairment of brain energy and development of brain edema appear to be central in the pathogenesis of encephalopathy [117, 118]. Inflammation plays a greater role in the pathogenesis of HE in patients with ACLF than in patients without ACLF and is associated with a cytokine storm. Recent data also suggest that neuro-inflammation may have a significant role in brain disturbance [117]. Cerebral edema has been observed in ACLF, and even low cerebral edema can be detected by proper CT and MRI. Vasogenic cerebral edema as detected by advanced MRI techniques like magnetization transfer ratio (MTR), diffusion tensor imaging (DTI) and diffusion weighted imaging (DWI) is common, but rarely requires specific treatment [118]. Using advanced MRI techniques, Gupta et al. demonstrated presence of cerebral edema increases with severity of ACLF. Correlation between mean diffusivity (MD) values and IL-6 levels suggest pathogenic role of inflammation in cerebral edema. MELD score and MD values in frontal white matter have prognostic significance in ACLF [118]. Ammonia, systemic inflammation and oxidative stress are key factors in the pathogenesis of HE, which may be modulated by glutaminase gene alteration or by the presence of spontaneous shunts [118].

Management of HE in hospitalized patients requires admission to the ICU and includes—(1) identification and treatment of precipitating factors, including infections and (2) specific measures for decreasing hyperammonemia and systemic inflammation. High volume plasmapheresis or albumin dialysis and identification and embolization of portosystemic shunts may be required in refractory patients. Recently analyzed AARC data showed that ammonia was significantly and persistently high in patients with grade III and IV HE (*p* < 0.001). l-Ornithine l-Aspartate (LOLA) for Hepatic Encephalopathy has a conflicting role as far as HE and ammonia reduction in patients of cirrhosis is concerned. Few recent meta-analysis showed a positive role of LOLA in reduction of ammonia as well as improvement in encephalopathy. Hence, LOLA can be considered as a potentially beneficial therapy for ACLF patients with HE and/or hyperammonemia [119]. However, a large prospective study would be needed. Emerging therapies include therapy for circulatory dysfunction and correction of hyponatremia [120].

Ammonia is a simple surrogate marker for HE in ACLF and correlates with severity of HE/cerebral failure. In the large AARC database, arterial ammonia levels were analyzed with respect to disease severity and outcome. At baseline, ammonia was significantly high in patients with cerebral dysfunction (HE grade I–II) and cerebral failure (HE grade III–IV). The patients who showed improvement in HE grades at day 4 and day 7 showed significant reduction in plasma ammonia level [121]. Dynamic change in ammonia level correlates well with clinical course of HE. However, ammonia-targeted therapy needs further trials and validation.

**Recommendations**

5.2. **Hepatic encephalopathy in ACLF.**
5.2.1. HE, including grade 1-2 HE (organ dysfunction) and grade 3-4 HE (organ failure), is present in about a third of the ACLF patients (2, B).
5.2.2. HE with all grades of severity and independent of other organ failures is associated with increased mortality; the mortality is higher in grade 3–4 (organ failure) compared with grade 1–2 HE (organ dysfunction) [1, B].
5.2.3. Inflammation plays a major role in the pathogenesis of HE in patients with ACLF and is associated with cytokine storm [1, B].
5.2.4. Management of HE in hospitalized patients requires admission to the high dependency or intensive care unit and includes—(1) identification and treatment of precipitating factors, including infections, and (2) specific measures for decreasing hyperammonemia and systemic inflammation. Large volume plasmapheresis or albumin dialysis and identification and embolization of portosystemic shunts may be required in refractory patients [1, C].
5.2.5. Ammonia is a simple surrogate marker for HE in ACLF and correlates with severity of HE/cerebral failure [2, B].
5.2.6. Dynamic change in ammonia level correlates well with clinical course of HE [2, B].
5.2.7. Ammonia-targeted therapy needs further trials and validation [2, B].
5.2.8. Lactulose, rifaximin, NH3-lowering strategies remain the main therapy for HE in patients with cirrhosis (1, B).

#### Coagulation in ACLF

In ACLF, alterations in primary and secondary hemostasis result in rebalanced coagulation, which leads to either bleeding or thrombotic episodes [122–125]. In addition, organ failures in ACLF may further disturb cirrhotic hemostatic imbalance. These include circulatory dysfunction [123], systemic inflammatory response syndrome [124, 125], sepsis, endogenous heparin-like substances or heparinoids [126] and renal dysfunction [127, 128].

Coagulation system in liver diseases is usually assessed by INR and platelet counts. Study by Premkumar et al. [129] and Blasi et al. [130] suggests that the ACLF is a hypocoagulable and hypofibrinolytic disorder and development of SIRS [131, 132] and sepsis further increases the hypocoagulability in these patients with increased chances of coagulopathic bleeding. Indirect evidence suggests that the endogenous heparinoids in ACLF patients with SIRS and sepsis could induce increased hypocoagulable state [130]. Conventional tests are insensitive to assess the complex coagulopathy in ACLF can be further complicated by SIRS and sepsis. The development of SIRS by day 7 further increases the INR (*p* < 0.001); however, no effect on platelets has been noted. ACLF cases with sepsis at presentation show increased INR and low platelets. Studies indicate the decline of platelets in first week associated with increased chances of organ failure and short-term mortality [131]. Acute variceal bleed at the time of presentation did not have association with baseline platelet counts; however, the INR was significantly higher in bleeders than non-bleeders in the recently analyzed AARC data.

PT-INR or prothrombin times is useful for prognostication, but are insensitive for detection of coagulopathy. More evidence-based algorithmic approach is needed for diagnosis and management of ACLF-induced coagulopathy.

**Recommendations**

5.3. **Coagulation in ACLF.**
5.3.1. ACLF is a hypocoagulable state and this can get escalated with the development of SIRS and sepsis [2, C].
5.3.2. Traditional coagulation measures, including prothrombin time (PT), activated partial thromboplastin time (aPTT), international normalized ratio (INR), fibrinogen levels and bleeding time (BT) do not measure bleeding risk in ACLF [2, B].
5.3.3. Coagulopathy assessment and management in ACLF should be guided based on global coagulation assessment system [ROTEM/TEG/SONOCLOT] [2, C].
5.3.4. Patients need to be characterized as bleeding or thrombosis phenotype by clinical assessment of major bleeding and d-dimer assay, respectively [2, C].
5.3.5. Global viscoelastic tests (TEG/Sonoclot/ROTEM) provide a more physiologic assessment of coagulation and should be considered to guide blood transfusion requirements in liver transplantation [1A] and other major surgery [2B] and invasive procedures [2C]. Application of global viscoelastic testing with ACLF in the ICU setting requires more data [2C].

#### Portal and systemic hemodynamics in ACLF and variceal bleed

Portal hypertension in liver disease is associated with both structural damage, which is the irreversible component, and dynamic component that includes increase in cytokine production, endothelial dysfunction, impaired vasorelaxation, and impaired vascular relaxation, which may be reversible component in the pathophysiology of portal hypertension after recovery. Thus, increased portal pressure in ACLF not only contributes to variceal bleeding but also to development of rapid onset ascites and other systemic complications including organ failures of ACLF. Majority of the patients with ACLF die during the first 45 days (median time to death 15 days) since the diagnosis [132]. ACLF patients have been shown to have much higher HVPG as compared to compensated cirrhotics. Among survivors of ACLF, complications such as ascites, coagulopathy gradually regress by 3 months [133]. It is thus likely that in patients with ACLF, the portal pressure gets acutely elevated and, after recovery, hepatic inflammation and cytokine levels decrease, which leads to improvement in hepatic as well as systemic hemodynamics. This was also indicated in a recent Asia Pacific multi-center study that higher cardiac output correlated with 30-day mortality (*p* < 0.019) and higher HVPG was associated with increased risk of variceal hemorrhage and mortality at 30 (*p* < 0.02) and 90 days (*p* < 0.001) [133]. Notably, these features were more pronounced in alcoholic hepatitis patients [134]. Choudhury et al. showed that baseline HVPG and mean pulmonary artery pressure were independent predictors of three-month mortality in ACLF [135].

##### Variceal progression and role of pharmacotherapy in ACLF

The rapid development of varices and bleeding is a matter of great concern in ACLF patients. It is important to determine the need of acute portal pressure reduction in these patients. It is likely that the patients would benefit from portal hypertension-reducing drugs like beta-blockers, especially in the acute phase of portal hypertension. NSBB have beneficial effects on the severity of portal hypertension, which requires both the beta-1 and beta-2 actions of the drug to ameliorate splanchnic vasodilation and high cardiac output.

Patients on NSBB had less severe grade of ACLF and a slower progression of ACLF during the study period. Patients who were receiving NSBBs in the previous 3 months and discontinued NSBBs (*n* = 78) after development of ACLF had a higher mortality (37% vs. 13%) and the main difference between those who discontinued or did not discontinue BB was the presence of circulatory dysfunction (hypotension requiring pressers) and respiratory failure. In another RCT comparing carvedilol with placebo in patients with ACLF (defined by APASL criteria) with either no or small esophageal varices and no contraindication to carvedilol use, carvedilol was found to reduce mortality, development of SBP and AKI at week 4 [135]. Thus, it is clear that patients should be continued on NSBBs, if feasible, even if ACLF develops [136].

Regarding the safety and radiation exposure of technical procedures for hepatic hemodynamics, Hari et al. studied the safety profile of HVPG measurement prospectively [137]. Accordingly, HVPG procedure showed a good safety profile and the radiation exposure was in most of the cases low. However, the HVPG measurement is invasive and difficult for routine clinical practice. Therefore, non-invasive surrogates that correlate well with invasive HVPG measurements are urgently needed in patients with ACLF [137–139].

##### PICD incidence, presentation, diagnosis and management in ACLF

Ascites is one of the syndrome defining components and in the AARC study, about 91% patients had ascites at presentation. About one-third of the patients presenting with ACLF do require paracentesis for severe grade ascites [140]. The presence of ascites in ACLF is different in many aspects from decompensated cirrhosis or AD [141]. Development of PICD in ACLF is associated with very high mortality. Albumin infusion was shown to significantly reduce mortality. Albumin infusion also helped in reducing to nearly half the incidence of development of new complications such as hyponatremia, hepatic encephalopathy and acute kidney injury. A high PRA level in ACLF patient reflects state of severe systemic inflammation, high portal pressure and systemic circulatory dysfunction [142, 143].

All precautions and monitoring including plasma renin activity are needed in undertaking ascetic tap in ACLF patients. Ascites in ACLF is part of acute portal hypertension and large volume paracentesis significantly alters the hemodynamics. Pharmacological agents, such as use of vasoconstrictors, should be studied to reduce the incidence of PICD in ACLF patients.

**Recommendations**

5.4.1. *Systemic, hepatic and pulmonary hemodynamics in ACLF.*
5.4.1.1. Baseline HVPG is an important predictor of mortality in ACLF (2, B).
5.4.1.2. The reduction in HVPG significantly influences the management of ACLF (2, C).
5.4.1.3. The safety and standardized procedure of HVPG measurement should be emphasized. Non-invasive surrogates of HVPG need to be investigated in ACLF (1, C).
5.4.2. *Variceal progression in ACLF and role of preemptive BB therapy.*
5.4.2.1. Increased portal pressure in ACLF can not only contribute to variceal bleeding but also other systemic complications and organ failures in ACLF [1, A].
5.4.2.2. NSBBs may reduce systemic inflammation and may have beneficial effects in ACLF patients over and above their portal hemodynamic effects [2, A].
5.4.2.3. ACLF patients should be started or continued on NSBBs, if there are no contraindications [2, B].
5.4.3. *PICD incidence, presentation, diagnosis and management.*
5.4.3.1. PICD is a result of significant derangement of systemic and splanchnic hemodynamics with a decrease in effective arterial blood volume, which is triggered by large volume paracentesis (> 5 L) [1, A].
5.4.3.2. PICD occurs in about 80% of ACLF patients when a large volume paracentesis is performed without additional therapeutic management. However, the incidence is reduced when volume expansion with albumin is used [1, A].
5.4.3.3. Terlipressin, a vasopressin analog, is partially effective and has been shown to have a synergistic effect with albumin in preventing PICD [1, B].
5.4.3.4. Non-selective β-blockers, such as propranolol, have been suggested to increase the risk of PICD and mortality in cirrhotic patients with refractory ascites. There is lack of data in patients with ACLF [2, B].

### Prognostic models and disease severity scores for ACLF

#### Prognostic models and disease severity scores

The disease severity assessment is needed for prognostication and to guide the therapy [144–146]. Disease severity scores such as model for end-stage liver disease (MELD) have been considered for organ allocation. However, MELD score does not take into account cerebral, circulatory and/or respiratory failures, thus giving no priority to patients with ACLF [147]. The various ICU scores like Sequential Organ Failure Assessment (SOFA), Acute Physiology and Chronic Health Evaluation (APACHE II) have also been evaluated for ACLF patients [147]. A study by Garg et al. in 2012 from New Delhi showed the predictability of these scores and also the relevance of one, two or more organ failures [28]. Subsequently, CLIF consortium has developed CLIF-SOFA score for assessing disease severity and prognostication in ACLF [11]. We had earlier shown that patients of ACLF have a high mortality in the presence of HE and hyponatremia in addition to high MELD, APACHE II and SOFA scores [28], necessitating inclusion of these parameters. A clinical event like HE or the laboratory parameters like bilirubin, creatinine, INR, serum sodium, plasma lactate or the liver histopathology reports and various disease severity scores do give near accurate prognostication.

Furthermore, the available prediction scores have been validated at baseline, but none has been evaluated in a dynamic manner for prognostication in ACLF patients. The severity of ACLF, rapid progression, development of sepsis and subsequent multi-organ failure (MOF), poor outcome with liver transplantation at the onset of MOF needs dynamic monitoring. Recent studies support for developing a dynamic model that could predict the outcome and appropriate time for LT. Chan et al. [147] in 149 patients showed that APACHE II scores ≥ 12 and MELD scores ≥ 28 after the first week of treatment were independent predictors of mortality. Mathurion et al. [148] showed that in the absence of response to corticosteroid for AH as assessed by Lille score on day 7 and consideration of early LT lead to significant cumulative 6-month survival rate (77 ± 8% vs. 23 ± 8%, *p* < 0.001). In large UK and US cohort of severe autoimmune hepatitis showed that not the baseline MELD/UKELD but the use of corticosteroid and no improvement in MELD/UKELD scores within 7 days had a poor outcome and suggested early consideration of other strategies including liver transplant [149–152]. A dynamic model that could predict the reversibility or need for liver transplant is urgently required. Early prediction of transplant-free survival, decision for transplant before onset of sepsis or multi-organ failure and prioritization for liver transplant could help improve the outcome of these patients.

#### Organ dysfunction and organ failure in ACLF for early risk stratification

The CLIF-SOFA and the CLIF-OF (Organ Failure) scoring and the cutoff were developed arbitrarily and included patients with hepatic and non-hepatic insults [11]. The organ failure was solely derived based on a consensus opinion by the experts [11]. The score is a bit cumbersome and becomes predictive of mortality only when extrahepatic organ failures are included. Earlier studies [28] and a recent study [152] showed that a simple score considering only the number of organ failures is easier to recall in predicting mortality in ACLF patients. The recently established and evaluated TPPM model in HBV ACLF patients from a single center large cohort, which displayed a superior predictive ability when compared with MELD and MELD-Na models [n-153]. TPPMs used TBIL, INR, HBV DNA and complications as parameters [153, 154]. Based on current multinational cohort, TPPMs demonstrated superiority to CLIF-C OFs, CLIF-C ACLFs, MELDs and MELD-Na in predicting 28-day and 90-day mortality in cirrhotic HBV-ACLF patients [155].

**Fig. 4:**
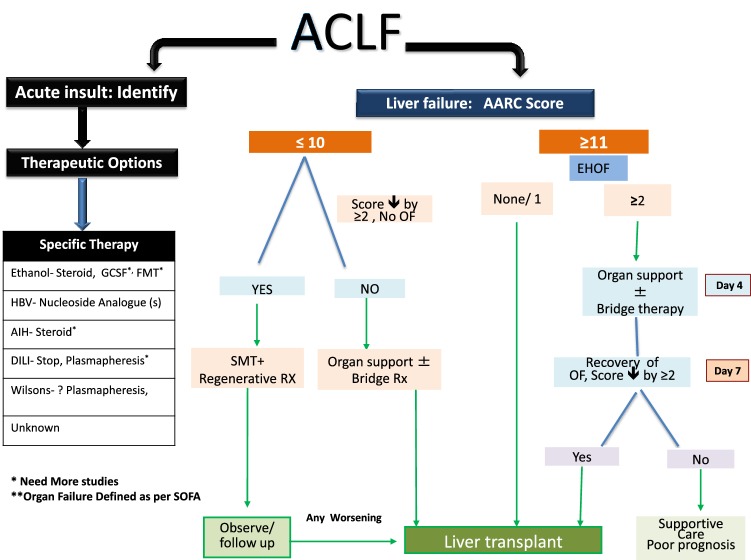
Algorithm for management of ACLF. The algorithmic approach to ACLF is highlighted based on the severity of liver failure, acute etiology and specific therapy and dynamic disease course. The specific treatment initiated, but if the disease severity is more, i.e., AARC Score (consideration of bilirubin, creatinine, INR, lactate and HE grade) 11 or more the response is poor with best medical supportive car; hence, early consideration for liver transplant should be done, whereas other group needs to be seen for 4–7 days with specific therapy and standard medical therapy. Any deterioration or AARC score 11 or more needs to consider LT. The presence of extrahepatic organ failure needs to be managed, and optimization and improvement need to be correlated with over all recovery else poor prognoses to be considered

**Fig. 5:**
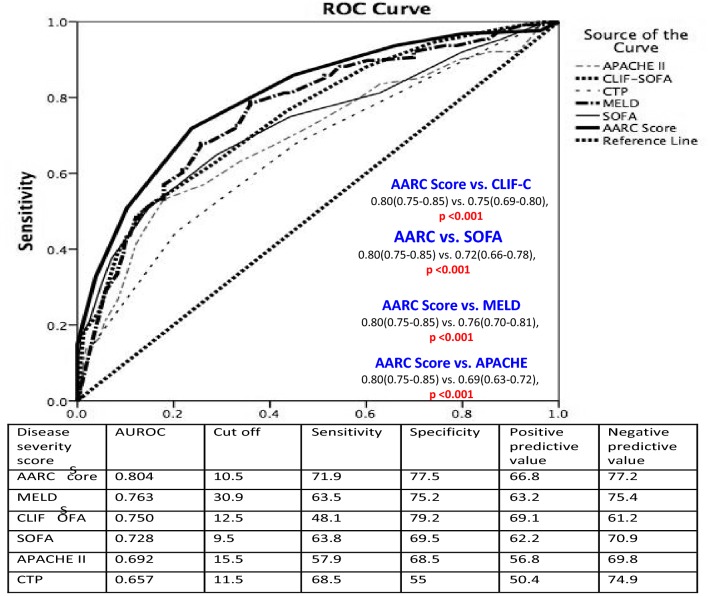
Comparison of AARC score against other disease severity score

The AARC score, as mentioned above, provides a physician a window to decide early and explore definitive therapies including liver transplantation (Table 4). A patient in whom the AARC score increases from 5 or 6 to 11 and above (change in grade of liver failure from I to III) at day 4 and day 7 increases the mortality significantly and a need for emergent transplantation in patients who fulfill the criteria. At the same time, the persistence of grade I or II liver failure till day 7 predicts improved survival, and a possibility of conservative treatment to be effective [3] (Fig. 4). AARC score has been compared with other scores for assessing the severity of ACLF such as SOFA, CLIF-SOFA, and MELD. The AARC score has been found to be superior to the other scores [3] (Fig. 5).

**Table 4:** AARC score and ACLF grade

AARC score					
Points	Total Bilirubin (mg/dl)	HE Grade	PT-INR	Lactate (mmol/l)	Creatinine (mg/dl)
1	< 15	0	< 1.8	< 1.5	< 0.7
2	15–25	I–II	1.8–2.5	1.5–2.5	0.7–1.5
3	> 25	III–IV	> 2.50	> 2.5	> 1.5
Minimum-5, Maximum-15					

AARC-ACLF grade	
Grade	Score
I	5–7
II	8–10
III	11–15

**Recommendations**

6.0. **Prognostic models and disease severity scores for ACLF.**
6.1. AARC score and other prognostic models.
6.1.1. AARC score is a very good prognostic model for the severity assessment and has been adequately validated. It has been proven to be superior to MELD/MELD Na., CLIF-SOFA and SOFA scores for patients with ACLF [2, A].
6.1.2. The cumulative mortality increases with rise in AARC score in the first week in Grade I (5–7), II (8–10) and III (11–15), respectively [2, A].
6.1.3. Treatment recommendations for ACLF should be based on AARC score. A trend of AARC score within first week can predict the need of liver transplant. Score of  < 10 at presentation or decrease in score below 10 by the end of first week is associated with higher chance of survival [1, B].
6.1.4. Patients with AARC Score > 10 should be listed for LT. Transplant evaluation based on the AARC score needs prospective studies [2, B].
6.1.5. AARC-ACLF score be estimated at admission in patients diagnosed to have ACLF as per AARC-ACLF definition [1, B].
6.1.6. AARC-ACLF score should also be estimated on Day 4 and Day 7 to predict the course of illness and prognosis [2, A].
6.1.7. The TPPM model has a superior predictive value for HBV ACLF outcomes than MELD and CLIFF-SOFA models (2, C).
6.1.8. AARC score holds good in predicting the outcome in critically ill ACLF patients [1, C]).
6.1.9. Lactate should be used for defining the severity of the ACLF syndrome. Lactate clearance compared to baseline lactate is a better indicator of patient status [1, C].
6.1.10. There is limited role of Renal Replacement Therapy (RRT) & Extra Corporeal Liver Support System (ECLS) to treat hyperlactetemia [2, B].

6.2. *Organ dysfunction and organ failure in ACLF for early risk stratification.*
6.2.1. Organ dysfunction and failure should not be used in the definition, but for prognostication in patients with ACLF [1, A].
6.2.2. Liver failure should be defined by a combination of bilirubin and INR and should be classified as mild/moderate and severe based on AARC as all patients have liver failure [1, B].
6.2.3. Liver, kidney and brain remain organs of utility in patients with ACLF [1, B].
6.2.4. Organ dysfunction and failure for brain should be based on AARC score [1, C].
6.2.4. Respiratory and circulatory failure may be considered as organs of futility in patients with ACLF which may contraindicate liver transplant [1, C].
6.2.5. For the diagnosis of organ dysfunction for kidneys in patients with ACLF as for patients with cirrhosis, AKIN criteria should be used [1, A].
6.2.6. Kidney failure should be defined based on serum creatinine above 1.5 mg/dl as per the AARC score [2, B].
6.2.7. The mortality in ACLF increases with the number of organ failures (1, C).

### Treatment of ACLF

#### Antiviral strategies in ACLF HBV reactivation

The presence of high HBV DNA [> 10(5) copies/ml/or > 2 × 10 (4) IU/ml] is highly sensitive and specific for the diagnosis [152]. Early and rapid reduction of HBV DNA is the essence of therapy [153]. Several studies have indicated that if the reduction in DNA of > 2 logs could be achieved within 2 weeks, the survival could be improved. This could be related to suppression of hepatocellular necrosis and cytokine release [154].

Besides patients who present with ACLF, it is worthwhile that prophylactic therapy should be considered for HBsAg-positive patients undergoing chemotherapy [155]. There are insufficient data to recommend antiviral therapy for HBsAg-negative and anti-HBc-positive patients with possible reactivation of occult HBV infection [156, 157].

**Recommendations**

7.1. *Antiviral strategies in ACLF HBV reactivation.*
7.1.1 Nucleos(t)ide analogs should be started immediately in all HBV-infected patients at presentation while waiting for confirmation by HBV DNA level. Potent antiviral drugs, such as tenofovir, tenofovir alafenamide or entecavir, should be used [2,B].
7.1.2. Assessment of reduction of HBV DNA level at day 15 after nucleos(t)ide analogs is encouraged; if < 2 log reduction, it suggests poor prognosis (2, B)..

### Liver transplantation

A characteristic feature of ACLF is its rapid progression, the requirement for multiple organ support and a high incidence of short- and medium-term mortality of 50–90%. The 28-day mortality rate was 15 times higher in patients with ACLF as compared to other chronic liver disease (CLD) patients [1–11, 158]. Patients with ACLF are susceptible to infection and early transplant-free survival is very low [159, 160]. Patients who develop infectious complications (particularly pneumonia and/or sepsis) and patients who receive renal replacement therapy or mechanical ventilation are less likely to undergo liver transplantation. Established sepsis/MODS precludes transplant and is associated with poor outcome. Both deceased and living donor transplants are viable and very useful options with very good results [161]. Liver transplant survival has been shown to be above 90% in patients with HBV reactivation [162].

Most important aspect in managing this group of sick patients is to decide the time frame and an algorithm. If the patient is too sick from the beginning and needs early LT without waiting for spontaneous recovery, this should be decided soon enough. On the other hand, patients who are salvageable and need time for recovery should be monitored closely in a time frame before deterioration so that they either recover or can undergo LT at an appropriate time. The third group is of those who need bridge therapy observed while on supportive care and bridging therapy, to define non-response and emergency LT or for transplant-free survival.

Every ACLF patient at admission needs to be assessed for disease severity score, presence of SIRS with or without sepsis, HE and number of organ dysfunction/failure. The baseline MELD > 28, AARC Score > 10, advanced HE in the absence of overt sepsis or multi-organ failure can be considered for early LT [3]. In the absence of LT option, these patients can be offered early bridge therapies in the form of therapeutic plasma exchange and liver dialysis and the response could be evaluated by end of first week and the possibility of being listed for LT or awaiting spontaneous recovery [3, 163]. The maximum recovery from organ failure, resolution of sepsis and eligibility for LT was observed in the first week [3]. The SIRS and/or sepsis and development of AKI occur by days 7–15; hence, the interventions like prophylactic antibiotics, periodic septic screening with the help of biomarkers and avoidance of nephrotixic drugs were needed [77].

ACLF is characterized by rapid downhill course with extrahepatic organ failure and high short- and medium-term mortality ranging from 34 to 51% [1]. Although many prediction models of early transplantation listing exist, none reliably predicts chances of reversibility of ACLF. A recent study showed that the ACLF patients develop SIRS and sepsis within 7 days of hospitalization [77]. Lin et al. [164] and Pamecha et al. [165] proposed serial assessment of these groups of patients in the first week of hospitalization for prioritization for liver transplantation. However, being sick, often critically ill and admitted to ICUs, rapid progression of liver failure and onset of multi-organ failure, transplantation was feasible in ~ 25% of patients [165]. Recently, a study showed that the LT waiting list mortality in ACLF patients is around 67% and is predominantly due to sepsis, respiratory failure with mechanical ventilation, high vasopressor requirement and need of RRT [165]. Though objective criteria were not used to define eligibility for LT, patient characteristics that were reported to consider an ACLF patient unfit were (1) sepsis with 2 or more organ failures or uncontrolled sepsis [166], (2) advanced azotemia, i.e., serum creatinine > 4 mg/dl or increase in creatinine by 300% from baseline or the need of Renal Replacement of therapy [167], (3) Respiratory failure [severe ARDS defined by a *P*/F ratio < 150] or HE requiring ventilator support > 72 h [168] (4) 4 or more organ failures anytime, (5) active gastrointestinal bleeding, and/or (6) hemodynamic instability requiring > 3 mg/h noradrenaline [169].

On the other hand, in the absence of liver transplantation, the outcome is dismal. In fact, liver transplant is potentially the only curative treatment option with good outcome, irrespective of etiology in this cohort. An analysis of 1021 patients from AARC cohort suggested MELD or MELD with HE is a good prediction model for making decision for LT. A patient with MELD > 27 needs listing, a score of 30 or above with presence of encephalopathy or new onset HE, bilirubin of > 22 mg/dl with INR > 2.5 and grade III-IV HE is associated with 80% mortality within 28 days and persistence of the same till day 4 is associated with mortality close to 100%. Hence, these patients need emergent LT either at baseline or upon no improvement within day 4–7 of hospitalization [163]. So first week of presentation in ACLF is crucial [77, 170]. This concept is supported by a window of 1 to 2 weeks, i.e., being sick and with no improvement by supportive care [171].

Disease severity scores such as MELD have been considered to determine organ allocation. This disease severity score has not taken into consideration of cerebral, circulatory and respiratory failure and does not offer any priority despite being sick [172, 173]. The recently published dynamic AARC model consisting of bilirubin, creatinine, INR, grade of hepatic encephalopathy and plasma lactate is a reliable model to predict the outcome at each time point, hence, the need of LT can be considered for prioritizing LT but further studies needed on this [3]. Emergency LT, promotion of live donor transplant and allocation priority in deceased donor setting needs consensus and further large size studies [77, 163, 170–176].

Sometimes, patients with ACLF have rapidly worsening liver and renal functions, needing quick decisions based on renal dysfunction. There is paucity of data on simultaneous liver and kidney transplant in this subset of ACLF patients [112].

**Recommendations**

7.2. Liver transplantation.
7.2.1. No validated criteria and scoring system for early and correct identification of patients with ACLF who would benefit from early liver transplantation [2, A].
7.2.2. LT should be offered early in the course of ACLF. When indicated, early liver transplantation avoids complications of sepsis and multi-organ failure [1, B].
7.2.3. SIRS, sepsis, HE, liver failure, extrahepatic organ dysfunction/organ failure are important prognostic factors [2, A].
7.2.4. Organ failure *per se* should not be a contraindication for liver transplantation, except if cardiac or pulmonary support is needed or there is rapidly progressive organ failure at day 4 or 7 [2, C].
7.2.5. APASL AARC model seems to be better in patient selection for liver transplantation as it enrolls patients early enough, before organ failure. However, the AARC score needs to be validated in large and varied populations and also its capability to predict post LT outcome [2, B].
7.2.6. Patients with HBV reactivation with intermediate MELD should be assessed for early transplant if cirrhosis, bilirubin > 10 mg/dL, PT < 40% and platelet < 100 × 10^9^/L [2, C].
7.2.7. Steroid ineligible patients with severe alcoholic hepatitis should be listed on priority for liver transplant. Selective use of liver transplantation can be lifesaving for medically refractory alcoholic hepatitis [1, A].
7.2.8. Liver transplantation should be reserved for severe alcoholic hepatitis patients with low risk of recidivism as assessed by a formal psychosocial evaluation [1, A].
7.2.9. Transplant evaluation can be started based on liver specific dynamic scores by the end of first week of medical treatment or earlier [2, B].
7.2.10. Allocation of cadaveric livers should depend on maturity of cadaveric program in the country [2, C].
7.2.11. Patients with advanced ACLF also have good outcome after LT [1, A].

#### Liver dialysis and emerging therapies in ACLF

The hepatocellular injury in ACLF is driven to a large extent by a “cytokine burst”, with elevated levels of multitude of cytokines, small molecular weight toxins, and vasoactive substances which are known to accumulate secondary to the failing liver [122]. There is an additional challenge of the injury due to endotoxin and metabolites released from gut bacteria. These toxins not only potentiate the hepatic injury but also deprive the liver of an environment, which is conducive for regeneration. The released toxins are responsible for the systemic inflammation, loss of adaptive and innate immunity and cause vital organ dysfunction, which affects all the major organs [174].

Extracorporeal liver support therapies are used to bridge the liver until recovery or liver transplantation in patients with ALF and ACLF. Various randomized controlled trials in patients with ACLF have shown improvement in HE, hepatorenal syndrome, circulatory dysfunction and immune dysfunction without improvement in transplant-free survival [175–182]. In the most recent meta-analysis and systematic review, no benefit of MARS treatment in reducing mortality as compared to SMT was noted [182]. Even though both these meta-analysis have the limitations of enrolling a heterogenous group of patients. However, contrary results were shown by systematic review by Kjaergard et al. where it was seen that ALS reduced mortality by 33% in patients with ACLF as compared to SMT [183, 184]. More recently, studies have shown that ALS could be an effective form of bridging therapy in patients with ACLF with high MELD scores awaiting liver transplantation and many believe that it is a futile exercise in the absence of liver transplant [185, 186]. These results have been substantiated by the recently published two large European randomized multicentric controlled trials, i.e., HELIOS (for Prometheus) [177] and RELIEF trial (for MARS) [176] which failed to show any benefit with these modalities on short-term transplant-free survival which was the primary end point of these studies. The foremost reason for no demonstrable survival benefit with the currently available artificial liver support systems is the functional incompetence as most of these provide only the detoxification function of the entire armamentarium of liver functions and thus incorporation of synthetic function by living hepatocytes, i.e., the “bioartificial liver” or therapies to potentiate hepatic regeneration look more realistic. The other major challenge that remains is to decide the timing of therapy so as to derive maximal therapeutic benefit, i.e., whether to consider it before or after the onset of sepsis because by the time multi-organ failure is manifest, the benefits of intervention with these devices are not to be expected.

In a large proportion of patients with ACLF, however, liver transplant is not feasible, due to lack of an organ, a donor, severity of the illness or other social challenges. There are few alternatives at present to liver transplant. There have been promising results of the use of growth factors in such patients. Garg et al. [28] have shown that granulocyte colony-stimulating factor (GCSF) can help in hepatic regeneration by mobilizing bone marrow-derived CD34 + cells. In addition, it significantly reduced the development of sepsis and subsequent multi-organ failure. These data were substantiated in another study from the East in patients with HBV-related ACLF [187–194].

However, despite the encouraging results and two randomized controlled clinical trials, it was felt that the use of these agents should be undertaken only under protocols and more data are required before recommending routine use of these agents.

**Recommendations**

7.3.1. Plasma exchange appears to be a promising and effective bridging therapy in patients with ACLF to liver transplant or spontaneous regeneration [1, C]
7.3.2. Plasma exchange can be safely undertaken in patients with ACLF in specialized liver units [2, B].
7.3.3. Plasmapheresis may be considered as a specific therapy for patients with Wilson’s disease and patients with severe flare of autoimmune liver disease (deemed unsuitable for steroids) [2, B].
7.3.4. Combination of PE with therapies to potentiate liver regeneration should be evaluated in patients with ACLF [2, C].

### Acute-on-chronic liver failure in children

An extensive PubMed search using keywords ‘Acute-on-chronic liver failure in children; ACLF in children; Pediatric acute-on-chronic liver failure; Pediatric ACLF’, returned only 5 studies, from 3 Indian centers [195–199]. The data on pediatric ACLF are limited and largely retrospective. Pediatric ACLF, although less commonly described in the literature, is not uncommon with a recent study demonstrating that around 14% of all CLD presented as ACLF [195]. ACLF was the first manifestation of a previously unknown underlying CLD in 75–100% cases as reported in some studies [196–198]. The combined data from the 3 centers showed that Wilson disease (41.2 – 45.7%) followed by autoimmune liver disease (17.6–41.9%) are the commonest CLD presenting as ACLF followed by cryptogenic cirrhosis (3.2–41.2%) [195–199]. The acute precipitating event was a hepatotropic viral insult (37–94.1%) in most. Flare of autoimmune liver disease (9.6–17%) and Wilson disease (0–27%), defined as ACLF in the absence of a recognizable acute event were also reported [196, 197], although the definition used is not established. In a study, no acute hepatic event was found in 29% of patients, a proportion of which could have been flare of Wilson disease [198]. As cholangitis is not currently accepted as parenchymal insult, which happens to be the most important event leading to decompensation in biliary atresia, the experts decided to exclude biliary atresia from ACLF definition.

Only 8.7% of the pediatric ACLF cohorts were ≤ 3 years. Metabolic liver diseases (MLD) account for majority of CLD in this age group. On analysis of MLD data, it was found that only 3/63 (4.8%) could fulfill the definition of ACLF but were labeled as ALF as there is some overlap in the 2 definitions [1, 199]. Children with MLD also failed to fulfill the definition of ACLF as a proportion of them either had longer jaundice to HE/ascites interval [200] or had anicteric liver failure. The common acute precipitating events of pediatric ACLF present less often before 5 years of age: acute hepatitis A in 15.7% (personal communication ILBS data) & drug hepatotoxicity: 27.8% [201].

#### Do we need a modified definition of pediatric ACLF?

There is no separate definition of pediatric ACLF. The major issues with the current definition in children are: (1) clinical identification of hepatic encephalopathy is often difficult and/or delayed specially those below 3 years of age, (2) clinical ascites may be difficult to identify especially in younger children with organomegaly, (3) some pediatric liver disease may present with liver failure without jaundice. The current ACLF definition requires jaundice to be followed by clinical ascites or HE within 4 weeks [1]. A delayed clinical identification or non-identification of HE/ascites will lead to the patient either being identified beyond the period of golden therapeutic window or not even diagnosed as ACLF. To circumvent the issue of difficult identification of HE, pediatric acute liver failure study group has defined acute liver failure as an INR greater than 2 regardless of the presence or absence of clinical HE [199]. Pediatric ACLF cohort at Institute of Liver and Biliary Sciences (ILBS) was evaluated to determine if the cutoff INR for defining ACLF can be increased to 2 regardless of the presence or absence of clinical HE. In the presence of HE, poor outcome was seen in 42.9% and 56.6% of those with INR between 1.5 and 2 (18/90) and those with INR ≥ 2 (74/90), respectively. As identification of HE is important but clinical identification often difficult, ammonia which has good correlation with HE [202] was evaluated as a surrogate marker. Ammonia performed poorly for diagnosis of HE with AUROC of 0.642 and an ammonia level more than 150 ug/dl diagnosed HE with 61.3% sensitivity and 70% specificity. EEG is another surrogate but is difficult to perform, not easily available and has not been standardized in children [203]. In the absence of a good surrogate, the experts agreed that clinical HE should continue to remain part of the definition. West Haven scale is used in older children, whereas modified HE assessment scale can be used for identifying and grading HE in children up to 3 years of age [199]. Detection of clinical ascites is difficult or delays diagnosis. Radiological ascites (mild to massive) from the ultrasound report and clinical ascites from the discharge summaries were compared in 127 children aged up to 3 years with CLD. The sensitivity of clinical examination to detect ascites was 71% with 29% being missed or identified later when the ascites increased further. Hence, the experts agreed to replace clinical ascites with clinical/radiological ascites in children.

#### Outcome and prognostication in pediatric ACLF

Theoretically, pediatric ACLF should have a better outcome than adults as the 2 commonest CLD, i.e., Wilson disease and autoimmune liver disease presenting as pediatric ACLF have specific medical therapy, better hepatic reserve (due to shorter duration of illness) and lesser incidence of co-morbidities. Outcome has been defined at different time points in the published pediatric literature [195–204]. Wilson disease and cryptogenic cirrhosis have poor prognosis with 48.8% and 30% survival, respectively. Those with acute HEV (50%), DILI (37.5%) and flare of Wilson disease (37.5%) have low survival. Half of the pediatric ACLF have one or more organ failure, with the commonest being cerebral and renal. Presence of ≥ 3 organ failures was associated with poor outcome. Outcome was poor in those with AKI and grade 3–4 HE. The presence of AKI increases the likelihood of death several folds [204]. Among the prognostic models evaluated in ACLF, APACHE-III, SOFA and CLIF SOFA score have been described in children [196, 197]. AARC score was recently shown to have excellent prognostic value in adult ACLF cohort [3]. Serum creatinine (SCr) value included in AARC score is unreliable as children have lower SCr, which is further accentuated due to malnutrition, sarcopenia and decreased endogenous production. A pediatric modification of AARC score was made replacing the absolute SCr with grades of AKI [204]. Both the AARC score (AUROC 0.945) and its pediatric modification (AUROC 0.951) were superior to the other prognostic scores in pediatric ACLF cohort. A cutoff score of 11 or more identified poor outcome with 87.5% sensitivity and 90.6% specificity. Poor outcome group showed a rise, while the good outcome group showed decline in AARC-ACLF score at day 4 (∆AARC-ACLF: Poor outcome: 1 ± 1.1 vs. Good outcome: − 0.6 ± 0.9, *p* < 0.0005) [204].

The proposal for a definition of ACLF in children by the APASL is the first such attempt to address the issue of ACLF in children. Hope this will be enriched by further large and multicentric studies in future.

**Recommendations**

8.0. **Acute-on-chronic liver failure in children**.
8.1. Pediatric ACLF is not uncommon (1, B).
8.2. The most common underlying liver diseases presenting as ACLF in children are Wilson disease and autoimmune liver disease (1, B).
8.3. The most common acute precipitating events are viral (HAV, HEV, HBV) hepatitis and flare of underlying disease/hepatotoxic drugs (1, B).
8.4. Standardized definitions of disease flare, as a precipitating event need to be further updated (2, C).
8.5. The existing definition of ACLF can be used to diagnose ACLF in children. However, there is an urgent need to generate more multicentric data from prospective studies.
8.6. Clinical diagnosis of hepatic encephalopathy, though difficult, is important for diagnosis/prognosis of pediatric ACLF. For diagnosis of HE in older children, West Haven scale can be used. For children less than 3 years, modified HE assessment scale can be used (2, C).
8.7. Clinical and/or radiological ascites can be used for defining ACLF in children (2, B).
8.8. Short-term (28-day) outcome is poor in approximately 33% of pediatric ACLF subjects (2, B).
8.9. One or more extrahepatic organ failures are seen in around half of children with ACLF (1, B).
8.10. Acute kidney injury and grade 3-4 HE are most common extrahepatic organ failures in pediatric ACLF.
8.11. Half the cases of ACLF at presentation have AKI. The presence of AKI increases the risk of poor outcome by several folds (2, B).
8.12. AARC-ACLF model is an easy, bedside, dynamic prognostic model for pediatric ACLF (B, 2).
8.13. A score more than or equal to 11 needs urgent listing and evaluation for liver transplant (2, B).
8.14. Pediatric modification of these scores may be useful (2, C).

## Conclusions

In summary, the field of ACLF has moved very rapidly in the past 5 years. The availability of large volume of published data from the East and the West has allowed reassessing the initial definitions. The need for having a well-defined homogenous population of patients, with well characterized acute and chronic insult and which would reflect the term acute-on-chronic liver failure, is at the core. Attempts to abrogate, ameliorate or reverse the ongoing injury would allow return of hepatic synthetic functions and reversal of the liver damage. Early predictors of mortality and non-reversibility of the condition would pave way to offer priority liver transplantation to such patients. An attempt to converge the thoughts from the East and West is possibly the only way forward to achieve more scientific and timely interventions for such seriously sick patients.

## Abbreviations

ACLF: Acute-on-chronic liver failure
DILI: drug-induced liver injury
CAM: Complimentary and alternative medicine
HDS: Herbs, drugs and supplements
AD: Acute decompensation
AIH: Auto immune hepatitis
BCS: Budd–Chiari syndrome
PVT: Portal vein thrombosis
AVB: Acute variceal bleed
PICD: Paracentesis induced circulatory dysfunction
RRT: Renal replacement therapy
LT: Liver transplant
SOFA: Sequential organ failure assessment
qSOFA: Quick sequential organ failure assessment
CANONIC-CLIF: Acute-oN-ChrONic LIver Failure in Cirrhosis
CLIF: Chronic Liver Failure Consortium
MELD: Model end-stage liver disease
TPPM: Tongji prognostic predictor model
