# Correction to: Acute-on-chronic liver failure: consensus recommendations of the Asian Pacific association for the study of the liver (APASL): an update

**DOI:** 10.1007/s12072-019-09980-1

**Published:** 2019-10-09

**Authors:** Shiv Kumar Sarin, Ashok Choudhury, Manoj K. Sharma, Rakhi Maiwall, Mamun Al Mahtab, Salimur Rahman, Sanjiv Saigal, Neeraj Saraf, A. S. Soin, Harshad Devarbhavi, Dong Joon Kim, R. K. Dhiman, Ajay Duseja, Sunil Taneja, C. E. Eapen, Ashish Goel, Q. Ning, Tao Chen, Ke Ma, Z. Duan, Chen Yu, Sombat Treeprasertsuk, S. S. Hamid, Amna S. Butt, Wasim Jafri, Akash Shukla, Vivek Saraswat, Soek Siam Tan, Ajit Sood, Vandana Midha, Omesh Goyal, Hasmik Ghazinyan, Anil Arora, Jinhua Hu, Manoj Sahu, P. N. Rao, Guan H. Lee, Seng G. Lim, Laurentius A. Lesmana, Cosmas Rinaldi Lesmana, Samir Shah, V. G. Mohan Prasad, Diana A. Payawal, Zaigham Abbas, A. Kadir Dokmeci, Jose D. Sollano, Gian Carpio, Ananta Shresta, G. K. Lau, Md. Fazal Karim, Gamal Shiha, Rino Gani, Kemal Fariz Kalista, Man-Fung Yuen, Seema Alam, Rajeev Khanna, Vikrant Sood, Bikrant Bihari Lal, Viniyendra Pamecha, Ankur Jindal, V. Rajan, Vinod Arora, Osamu Yokosuka, Madunil A. Niriella, Hai Li, Xiaolong Qi, Atsushi Tanaka, Satoshi Mochida, Dominic Ray Chaudhuri, Ed Gane, Khin Maung Win, Wei Ting Chen, Mohd. Rela, Dharmesh Kapoor, Amit Rastogi, Pratibha Kale, Archana Rastogi, Chhagan Bihari Sharma, Meenu Bajpai, Virender Singh, Madhumita Premkumar, Sudhir Maharashi, A. Olithselvan, Cyriac Abby Philips, Anshu Srivastava, Surender K. Yachha, Zeeshan Ahmad Wani, B. R. Thapa, Anoop Saraya, Shalimar, Ashish Kumar, Manav Wadhawan, Subash Gupta, Kaushal Madan, Puja Sakhuja, Vivek Vij, Barjesh C. Sharma, Hitendra Garg, Vishal Garg, Chetan Kalal, Lovkesh Anand, Tanmay Vyas, Rajan P. Mathur, Guresh Kumar, Priyanka Jain, Samba Siva Rao Pasupuleti, Yogesh K. Chawla, Abhijit Chowdhury, Shahinul Alam, Do Seon Song, Jin Mo Yang, Eileen L. Yoon, APASL ACLF Research Consortium (AARC) for APASL ACLF working Party.

**Affiliations:** 1 Department of Hepatology, Institute of Liver and Biliary Sciences, New Delhi, 110070 India; 2 Department of Hepatology, Bangabandhu Sheikh Mujib Medical University, Dhaka, Bangladesh; 3 Department of Hepatology, Medanta The Medicity, Gurgaon, India; 4 Department of Hepatology, St John Medical College, Bangalore, India; 5 Department of Internal Medicine, Hallym University College of Medicine, Seoul, South Korea; 6 Department of Hepatology, PGIMER, Chandigarh, India; 7 Department of Hepatology, CMC, Vellore, India; 8 Institute and Department of Infectious Disease, Tongji Hospital, Tongji Medical College, Huazhong University of Science and Technology, Wuhan, China; 9 Translational Hepatology Institute Capital Medical University, Beijing You’an Hospital, Beijing, China; 10 Department of Medicine, Chulalongkorn University, Bangkok, Thailand; 11 Department of Medicine, Aga Khan University Hospital, Karachi, Pakistan; 12 Department of Gastroenterology, Lokmanya Tilak Municipal General Hospital and Lokmanya Tilak Municipal Medical College, Sion, Mumbai, India; 13 Department of Gastroenterology, SGPGIMS, Lucknow, India; 14 Department of Medicine, Hospital Selayang, Bata Caves, Selangor, Malaysia; 15 Department of Gastroenterology, DMC, Ludhiana, India; 16 Department of Hepatology, Nork Clinical Hospital of Infectious Disease, Yerevan, Armenia; 17 Department of Gastroenterology and Hepatology, Sir Ganga Ram Hospital and GRIPMER, New Delhi, Delhi India; 18 Department of Medicine, 302 Millitary Hospital, Beijing, China; 19 Department of Gastroenterology and Hepatology Sciences, IMS & SUM Hospital, Bhubaneswar, Odisha India; 20 Asian Institute of Gastroenterology, Hyderabad, India; 21 Division of Gastroenterology and Hepatology, Department of Medicine, National University Health System, Singapore, Singapore; 22 Digestive Disease and GI Oncology Centre, Medistra Hospital, Jakarta, Indonesia; 23 Department of Hepatology, Global Hospitals, Mumbai, India; 24 Department of Gastroenterology, VGM Hospital, Coimbatore, India; 25 Fatima University Medical Center Manila, Manila, Philippines; 26 Department of Medicine, Ziauddin University Hospital, Karachi, Pakistan; 27 Department of Medicine, Ankara University School of Medicine, Ankara, Turkey; 28 Department of Medicine, University of Santo Tomas, Manila, Philippines; 29 Department of Hepatology, Foundation Nepal Sitapaila Height, Kathmandu, Nepal; 30 Department of Medicine, Humanity and Health Medical Group, New Kowloon, Hong Kong China; 31 Department of Hepatology, Sir Salimullah Medical College, Dhaka, Bangladesh; 32 Egyptian Liver Research Institute And Hospital, Cairo, Egypt; 33 Division of Hepatobiliary, Department of Internal Medicine, Faculty of Medicine, Cipto Mangunkusumo Hospital, Universitas Indonesia, Jakarta, Indonesia; 34 Department of Medicine, Queen Mary Hospital Hong Kong, The University of Hong Kong, Hong Kong, China; 35 Department of Pediatric Hepatology, Institute of Liver and Biliary Sciences, New Delhi, Delhi India; 36 Department of Hepatobilliary Pancreatic Surgery and Liver Transplant, Institute of Liver and Biliary Sciences, New Delhi, Delhi India; 37 Professor Emeritus, Chiba University, Chiba, Japan; 38 Department of Medicine, University of Kelaniya, Ragama, Sri Lanka; 39 Department of Gastroenterology, Ren Ji Hospital, School of Medicine, Shanghai Jiao Tong University, Shanghai, China; 40 CHESS Frontier Center, The First Hospital of Lanzhou University, Lanzhou University, Lanzhou, China; 41 Department of Medicine, Tokyo University School of Medicine, Tokyo, Japan; 42 Department of Gastroenterology and Hepatology, Faculty of Medicine, Saitama Medical University, Saitama, Japan; 43 New Zealand Liver Transplant Unit, Auckland Hospital, Auckland, New Zealand; 44 Yangon GI and Liver Centre, Pabedan, Yangon, Myanmar; 45 Division of Hepatology, Department of Gastroenterology and Hepatology, Chang Gung Medical Foundation, Linkou Chang Gung Memorial Hospital, Taoyuan, Taiwan; 46 Department of Liver Transplant Surgery, Dr. Rela Institute and Medical Centre, Chennai, India; 47 Department of Microbiology, Institute of Liver and Biliary Sciences, New Delhi, Delhi India; 48 Department of Pathology, Institute of Liver and Biliary Sciences, New Delhi, Delhi India; 49 Department of Immunohematology and Transfusion Medicine, Institute of Liver and Biliary Sciences, New Delhi, Delhi India; 50 Department of Gatroenterology, SMS Med College, Jaipur, India; 51 Division of Liver Transplantation and Hepatology, Manipal Hospitals, Bangalore, India; 52 The Liver Unit, Cochin Gastroenterology Group, Ernakulam Medical Centre, Kochi, India; 53 Department of Pediatric Gastroenterology, SGPGIMS, Lucknow, India; 54 Noora Hospital in Srinagar, Shrinagar, India; 55 Department of Gastroenterology and Pediatric Gastroenterology, PGIMER, Chandigarh, India; 56 Department of Gastroenterology and Human Nutrition, AIIMS, New Delhi, India; 57 Department of Gastroenterology, Hepatology and Liver Transplant, B L K Hospital, New Delhi, India; 58 Centre for Liver and Biliary Science, Max Hospital, New Delhi, India; 59 Department of Gastroenterology, Hepatology and Liver Transplant, Max Hospital, New Delhi, India; 60 Department of Pathology, GB Pant Hospital, New Delhi, India; 61 Department of Liver Transplant and Hepatobilliary Surgery, Fortis Hospital, New Delhi, India; 62 Department of Gastroenterology, GB Pant Hospital, New Delhi, India; 63 Department of Gastroenterology, Hepatology and Liver Transplant, Apollo Hospital, New Delhi, India; 64 Department of Hepatology, Sir H N Reliance Hospital and Research Centre, Mumbai, India; 65 Department of Gastroenterology and Hepatology, Narayana Hospital, Gurugram, India; 66 Department of Hepatology, Parimal Multi-Speciality Hospital, Ahmedabad, India; 67 Department of Nephrology, Institute of Liver and Biliary Sciences, New Delhi, India; 68 Department of Statistics and Clinical Research, Institute of Liver and Biliary Sciences, New Delhi, India; 69 Department of Hepatology and Gastroenterology, Kalinga Institute of Med Sciences, KIIT University, Bhubaneswar, India; 70 Department of Hepatology, Institute of Post Graduate Medical Education and Research, Kolkata, India; 71 Department of Internal Medicine, College of Medicine, The Catholic University of Korea, Seoul, South Korea; 72 Department Of Internal Medicine, Inje University College of Medicine, Busan, South Korea

## Correction to: Hepatology International (2019) 13:353–390 10.1007/s12072-019-09946-3

The article Acute-on-chronic liver failure: consensus recommendations of the Asian Pacific association for the study of the liver (APASL): an update, written by [Shiv Sarin], was originally published electronically on the publisher’s internet portal (currently SpringerLink) on June 06, 2019 without open access.

After publication in volume 13, issue 4, page 353-390 the author decided to opt for Open Choice and to make the article an Open Access publication. Therefore, the copyright of the article has been changed to © The Author(s) 2019 and the article is forthwith distributed under the terms of the Creative Commons Attribution 4.0 International License (http://creativecommons.org/licenses/by/4.0/), which permits use, duplication, adaptation, distribution and reproduction in any medium or format, as long as you give appropriate credit to the original author(s) and the source, provide a link to the Creative Commons license and indicate if changes were made.

The original article has been corrected.

